# Aurora-A-Dependent Control of TACC3 Influences the Rate of Mitotic Spindle Assembly

**DOI:** 10.1371/journal.pgen.1005345

**Published:** 2015-07-02

**Authors:** Selena G. Burgess, Isabel Peset, Nimesh Joseph, Tommaso Cavazza, Isabelle Vernos, Mark Pfuhl, Fanni Gergely, Richard Bayliss

**Affiliations:** 1 Department of Biochemistry, University of Leicester, Leicester, United Kingdom; 2 Cancer Research UK Leicester Centre, University of Leicester, Leicester, United Kingdom; 3 Cancer Research UK Cambridge Institute, Li Ka Shing Centre, University of Cambridge, Cambridge, United Kingdom; 4 Cell and Developmental Biology program, Centre for Genomic Regulation (CRG), Barcelona, Spain; 5 Universitat Pompeu Fabra (UPF), Barcelona, Spain; 6 Institució Catalana de Recerca i Estudis Avançats (ICREA), Barcelona, Spain; 7 Cardiovascular and Randall Division, King’s College London, London, United Kingdom; Heinrich-Heine-University, GERMANY

## Abstract

The essential mammalian gene *TACC3* is frequently mutated and amplified in cancers and its fusion products exhibit oncogenic activity in glioblastomas. TACC3 functions in mitotic spindle assembly and chromosome segregation. In particular, phosphorylation on S558 by the mitotic kinase, Aurora-A, promotes spindle recruitment of TACC3 and triggers the formation of a complex with ch-TOG-clathrin that crosslinks and stabilises kinetochore microtubules. Here we map the Aurora-A-binding interface in TACC3 and show that TACC3 potently activates Aurora-A through a domain centered on F525. Vertebrate cells carrying homozygous F525A mutation in the endogenous *TACC3* loci exhibit defects in TACC3 function, namely perturbed localization, reduced phosphorylation and weakened interaction with clathrin. The most striking feature of the F525A cells however is a marked shortening of mitosis, at least in part due to rapid spindle assembly. F525A cells do not exhibit chromosome missegregation, indicating that they undergo fast yet apparently faithful mitosis. By contrast, mutating the phosphorylation site S558 to alanine in TACC3 causes aneuploidy without a significant change in mitotic duration. Our work has therefore defined a regulatory role for the Aurora-A-TACC3 interaction beyond the act of phosphorylation at S558. We propose that the regulatory relationship between Aurora-A and TACC3 enables the transition from the microtubule-polymerase activity of TACC3-ch-TOG to the microtubule-crosslinking activity of TACC3-ch-TOG-clathrin complexes as mitosis progresses. Aurora-A-dependent control of TACC3 could determine the balance between these activities, thereby influencing not only spindle length and stability but also the speed of spindle formation with vital consequences for chromosome alignment and segregation.

## Author Summary

Maintenance of genomic fidelity depends on the faithful division of chromosomes by the mitotic spindle, a molecular system comprising microtubules and associated proteins. The timely establishment of a functional bipolar spindle requires co-operation between several assembly pathways, coordinated by protein kinases such as Aurora-A. A key substrate of Aurora-A in mitosis is TACC3. Phosphorylation of TACC3 triggers its binding to clathrin to form a complex that promotes crosslinking and stabilization of spindle microtubules. We identify a new binding interface between Aurora-A and TACC3 that is crucial for assembly of TACC3-clathrin complexes and therefore proper spindle localization of TACC3. Disruption of this interface results in an accelerated yet faithful mitosis. We conclude that mutual regulation between Aurora-A kinase and its substrate TACC3 constitutes a molecular switch between different spindle assembly pathways, thereby influencing the speed of spindle formation.

## Introduction

Formation of a functional mitotic spindle is a pre-requisite for equal distribution of chromosomes between two daughter cells. Mitotic spindles in animal cells consist of three major microtubule (MT) classes: centrosome-associated astral MTs, inter-polar MTs and kinetochore-fibres (k-fibres). During mitosis MTs are produced by multiple simultaneously acting pathways that include centrosomal MT nucleation, Ran-GTP-driven chromatin-dependent MT generation and MT-dependent MT amplification through the Augmin complex [1]. When present the centrosomes act as dominant sites of spindle pole formation, but in all animal cells MT motors and MT-associated proteins play important roles in stabilizing, organising and transporting MTs generated elsewhere in the cytoplasm for incorporation into the mitotic spindle [2,3]. Co-operation of these spindle assembly pathways seems important for the timely establishment of a bipolar mitotic spindle [4].

Aurora-A is a Ser/Thr protein kinase that regulates key mitotic events such as centrosome maturation, mitotic entry and spindle assembly [5,6]. In particular, Aurora-A function is essential for bipolar spindle formation; loss of Aurora-A kinase activity through gene targeting, RNA interference-mediated depletion or inhibition results in the assembly of characteristic monopolar spindles. A range of additional phenotypes such as short bipolar and multipolar spindles, MT hyper-stabilisation, centrosome and spindle pole fragmentation or chromosome alignment and segregation defects have also been reported in several model systems [7–12]. As a consequence of these spindle defects anaphase onset is delayed in many of these models by an active spindle assembly checkpoint (SAC), which ensures that all chromosomes have bi-oriented and aligned at the metaphase plate in a cell before sister chromatid separation is initiated [12,13]. Aurora-A exerts its control over spindle assembly through phosphorylation of several substrates, including TACC3 [14,15].

TACC3, an essential protein in mammals, is a member of the transforming acidic coiled coil (TACC) family of centrosomal proteins that bind MTs and interact with the MT polymerase ch-TOG, highly conserved from yeast to mammals [16–20]. TACC3 and its lower eukaryotic homologues, D-TACC in *Drosophila* and Maskin in *Xenopus laevis*, have been implicated in both centrosome- and chromatin-driven MT assembly pathways during mitotic spindle assembly [15,21–29]. Although the molecular details of TACC3 function remain poorly understood, reports tend to support a central role of TACC3 in promoting MT stability in mitosis [16]. In humans, Aurora-A phosphorylates TACC3 on three residues (S34, S552 and S558); these sites are conserved in Maskin and the S558 equivalent site is also present in D-TACC [26,27,30]. In mammalian cells, phosphorylation of S558 promotes accumulation of TACC3 on spindle MTs [7] and regulates its binding to clathrin, a protein with a well-established function in endocytosis [31–34]. TACC3 in a complex with clathrin and ch-TOG forms bridges between k-fibres, potentially cross-linking and stabilising these mitotic MT species [35]. Phosphorylated TACC3 binds to the ankle region of clathrin, generating a MT-binding surface that involves the coiled-coil region of TACC3 and the β-propeller domain of clathrin [36]. Intriguingly, this combined MT binding domain mediates association of the TACC3-clathrin-ch-TOG protein complex with MTs despite the high binding affinity of ch-TOG for tubulin and MTs [37]. TACC3 and ch-TOG can interact independently of Aurora-A, and it is thus possible that TACC3 exists both in TACC3-ch-TOG and TACC3-clathrin-ch-TOG complexes in mitotic cells [38]. TACC3 function has been implicated in many different tumour types [39]. In addition to various mutations and amplifications in the gene, oncogenic fusion products between FGFR3 and TACC3 have been identified in glioblastoma, bladder, lung and nasopharyngeal carcinomas [40–44]. These oncogenic fusion products contain the C-terminal TACC domain of TACC3 but lack the clathrin-binding domain inclusive of the Aurora-A phosphorylation site, indicating that these sequences might serve to keep TACC3 activity under check.

The activity of Aurora-A is controlled through phosphorylation of the activation loop on Thr288 [45,46]. In addition, several activating binding partners of Aurora-A have been identified, many of which are also substrates of the kinase [6]. In the case of TPX2, the mechanism of activation, and how this synergizes with phosphorylation of T288, has been resolved [47–49]. The first 43 amino acids of TPX2 stimulate Aurora-A activity, whether the kinase is phosphorylated or not. Thus, TPX2-binding and Aurora-A autophosphorylation work together to stabilize the activation loop in a fully ordered conformation similar to that observed in other Ser/Thr kinases, such as protein kinase A (PKA).

Although the function of Aurora-A phosphorylation on TACC3 has been extensively studied, the reciprocal effects of TACC3 on Aurora-A have not received attention. In this study, we investigated the molecular basis of the interaction between Aurora-A and TACC3. We discover a new Aurora-A binding site in TACC3 that has functions independent of the phosphorylation site. Mutation of either site results in a similar reduction of spindle localization of TACC3, but has distinct consequences for mitotic progression and chromosome segregation.

## Results

### TACC3 stimulates the activity of Aurora-A

**Fig 1:**
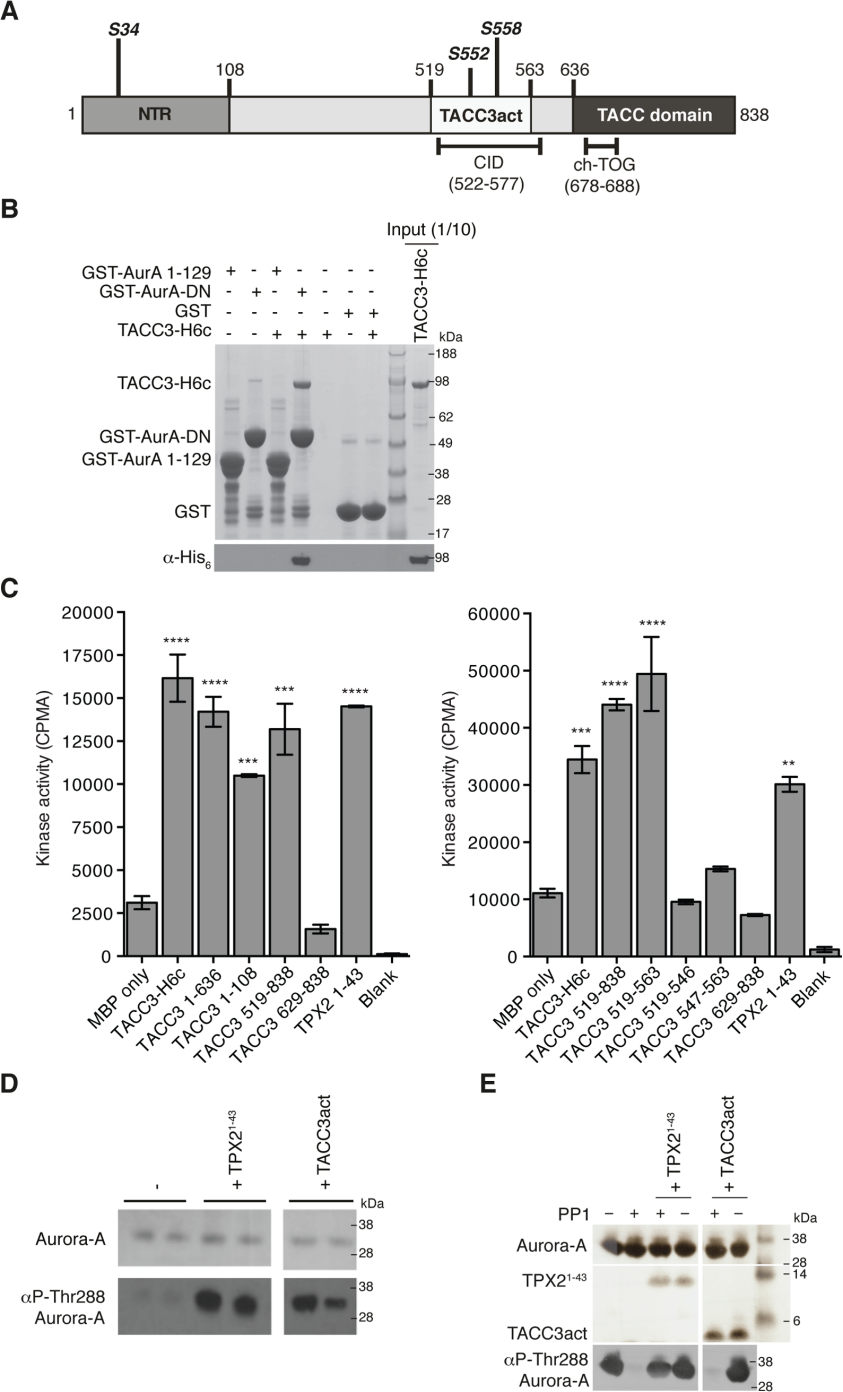
Biochemical characterisation of Aurora-A activation by TACC3. (A) The domain structure of human TACC3 is shown with conserved regions marked: N-terminal region (NTR, residues 1–108, coloured medium grey), Clathrin Interaction Domain (CID, residues 522–577, marked below), TACC domain (residues 636–838, coloured dark grey). Aurora-A phosphorylation sites are marked in bold italics. Known protein binding regions are marked below. (B) Co-precipitation assay between GST-AurA 1–129, GST-AurA-DN and TACC3-H6c. GST was used as a binding control. Reactions were analysed by SDS-PAGE (top panel). Binding of TACC3-H6c was confirmed by Western blot using an α-His_6_ antibody (bottom panel). (C) *In vitro* kinase activity assay of Aurora-A 122–403 in the presence of TACC3-H6c and TACC3 fragments. The known Aurora-A activator, TPX2^1-43^ was used as a positive control. Incorporation of ^32^P radioisotope into MBP was quantified by scintillation counting. Error bars represent the standard error for two independent reactions. ** = P<0.01, *** = P<0.001 and **** = P<0.0001 using one-way ANOVA with Dunnett's post-hoc test compared to the MBP only reaction. SDS-PAGE analysis of TACC3 proteins used in this assay is shown in S1A Fig. (D) Stimulation of Aurora-A 122–403 autophosphorylation by TPX2^1-43^ and TACC3act. Total Aurora-A is shown in the SDS-PAGE gel (top panel). Levels of phosphorylation were observed by Western blot using an antibody specific to Aurora-A phosphorylated on Thr288 (bottom panel). (E) Protection of Aurora-A 122–403 from dephosphorylation by PP1 in the presence of TPX2^1-43^ and TACC3act. Aurora-A, TPX2^1-43^ and TACC3act were resolved by SDS-PAGE (top panel). Aurora-A phosphorylation was detected by Western blot using a α-phosphoThr288 Aurora-A antibody (bottom panel).

TACC3 has three notable conserved regions: the N-terminal region (NTR) that includes S34, the clathrin interaction domain (CID) that includes S552 and S558, and the C-terminal TACC domain (Fig 1A). GST co-precipitation assays were performed to identify the TACC3 binding domain in Aurora-A (Fig 1B). Binding occurs between the catalytic domain of Aurora-A (residues 122–403, GST-AurA-DN) and TACC3-H6c. We investigated whether TACC3 influenced the activity of phosphorylated, active Aurora-A catalytic domain, as other substrates do, using an *in vitro* kinase assay to monitor the incorporation of ^32^P into a generic substrate (myelin basic protein, MBP), which was quantified using scintillation counting (Fig 1C). TACC3 enhanced Aurora-A activity by a factor of ~3, very similar to the effect of the first 43 amino acids of TPX2. We mapped the region of TACC3 responsible for Aurora-A activation using TACC3 fragments. The NTR stimulated Aurora-A activity, but to a lesser extent than the full-length TACC3. The TACC domain did not contribute to Aurora-A activation. The central region (residues 519–563) stimulated Aurora-A activity to an even greater extent than full-length TACC3, as was observed for TPX2^1-43^ versus full-length TPX2 [48]. We therefore focused on this region of TACC3, which includes most of the CID but lacks the di-leucine motif that is critical for clathrin binding [36]. To avoid confusion with the CID region of TACC3, we refer to this region as TACC3act hereafter.

In addition to the allosteric activation of phosphorylated Aurora-A, TPX2 regulates the phosphorylation of Aurora-A through two mechanisms: stimulation of autophosphorylation and protection from dephosphorylation by PP1. We therefore asked whether TACC3act modulates Aurora-A phosphorylation *in vitro*. Like TPX2, TACC3act stimulated autophosphorylation of initially unphosphorylated Aurora-A catalytic domain, as measured by western blotting using a phospho-specific T288 Aurora-A antibody (Fig 1D). Unlike TPX2, TACC3act did not protect initially phosphorylated Aurora-A catalytic domain from dephosphorylation by PP1 (Fig 1E). Although TACC3act resembles TPX2^1-43^, as both are relatively small protein fragments capable of stimulating Aurora-A activity, this result raised the question of whether they might have different binding sites on Aurora-A. We proceeded to characterize the interaction between the catalytic domain of Aurora-A and TACC3act.

**Fig 2:**
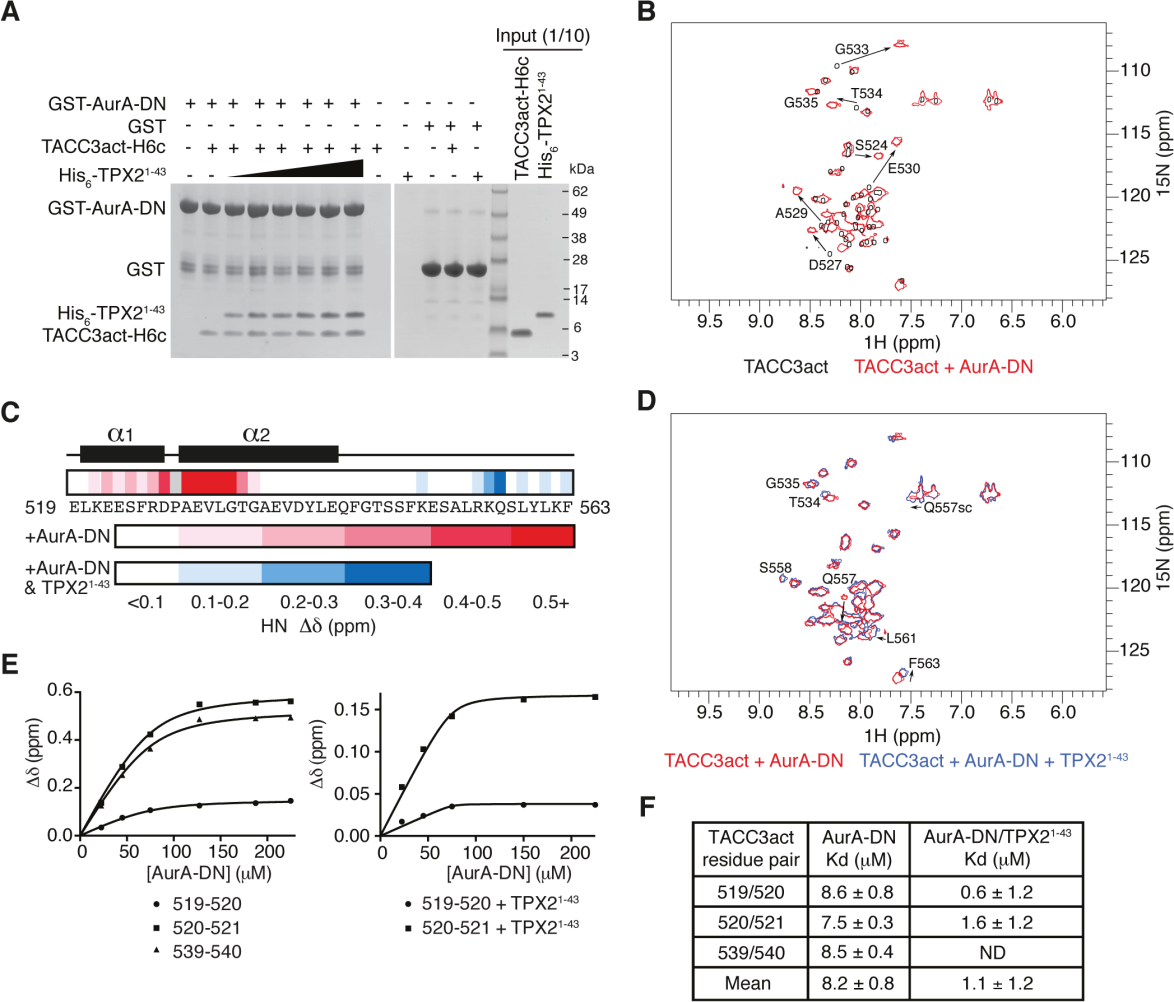
Biophysical characterisation of Aurora-A binding to TACC3act and comparison with TPX2^1-43^. (A) Co-precipitation assay between GST-AurA-DN and TACC3act-H6c and His_6_-TPX2^1-43^. 5 μM TACC3act-H6c was used in reactions with 1, 2, 5, 10, 20 and 50 μM His_6_-TPX2^1-43^ (black triangle). GST was used as a binding control. (B) ^1^H-^15^N HSQC spectra of ^15^N-labelled TACC3act in the absence (black) and presence (red) of AurA-DN. TACC3act residues are labelled and chemical shift changes observed on interaction with AurA-DN are marked with arrows. (C) Summary of NMR data mapped onto the primary sequence of TACC3act. Changes in backbone chemical shifts (Δδ) upon binding to Aurora-A are shown on a scale from low (white) to high (red), based on S1C Fig. Secondary structure content is derived from S2 Fig. Chemical shift changes in the presence of TPX2^1-43^ are shown on a scale from low (white) to high (blue) using data in S1D Fig. No data is available for P528 (coloured grey) because it lacks a backbone N-H. (D) ^1^H-^15^N HSQC spectra of ^15^N-labelled TACC3act in the presence of AurA-DN (red) and on the addition of TPX2^1-43^ (blue). TACC3act residues are labelled and chemical shift changes observed on interaction with TPX2^1-43^ are marked with arrows. (E) TACC3act chemical shift changes associated with increasing concentrations of AurA-DN were monitored in the absence (left) or presence (right) of TPX2^1-43^ and fit to Eq 1. (F) Binding affinity of TACC3act for AurA-DN in the absence and presence of TPX2^1-43^ as determined in Fig 2E. ND, not determined.

We investigated whether the interaction of TACC3act with Aurora-A was competitive with TPX2^1-43^ by GST co-precipitation assay. A catalytically inactive mutant (D274N) of the Aurora-A catalytic domain fused to GST was used as bait and we established a robust interaction with TACC3act, where TPX2^1-43^ was used as a positive control for Aurora-A interaction (Fig 2A). GST alone was used as a negative control to show that binding of TACC3act and TPX2^1-43^ to GST-Aurora-A was specific. We observed that TACC3act and TPX2^1-43^ were able to bind simultaneously to Aurora-A, with no apparent reduction in interaction when the concentration of TPX2^1-43^ was increased to a 10-fold excess over TACC3. Size exclusion chromatography (SEC) analysis of TACC3act and TPX2^1-43^ showed that the retention volume of the two activators is decreased in the presence of Aurora-A kinase domain, and that the three proteins co-eluted (S1B Fig). Taken together, these observations indicate simultaneous interactions of TACC3 and TPX2 with Aurora-A and suggest that the two activators have different, non-competitive binding sites on the kinase.

In brief, we have shown that TACC3 stimulates Aurora-A activity through a binding region, TACC3act (residues 519–563), which overlaps with the CID. We further investigated the molecular determinants of this interaction using structural biology approaches.

### NMR studies reveal two distinct Aurora-A binding sites in TACC3

We employed NMR spectroscopy to map the precise binding site of Aurora-A on TACC3act. Our first NMR experiments probed the changes in the chemical environment of the backbone residues of TACC3act in the presence or absence of unphosphorylated Aurora-A 122–403 D274N (AurA-DN; Fig 2B and S1C Fig). A ^1^H-^15^N HSQC spectrum of TACC3act alone showed reasonable peak separation (Fig 2B), and the backbone was assigned using CCPN analysis [50]. Secondary structure specific data were collected, which showed residues 520–527 and 529–542 on human TACC3 form a pair of nascent α-helices separated by P528 (Fig 2C and S2 Fig).

A ^1^H-^15^N HSQC spectrum of ^15^N-labelled TACC3act in the presence of AurA-DN showed that a subset of residues within TACC3act experience chemical shift perturbations when bound to Aurora-A (Fig 2B). The change in chemical shift of the backbone amide of each residue was measured and mapped onto the primary sequence of TACC3act (Fig 2C and S1C Fig). The majority of the chemical shift perturbations were restricted to the N-terminal region of TACC3act, between residues 521 and 535. The greatest values were found close to P528, at residues D527 and 529–533, consistent with a strong involvement of this region of TACC3 in binding to Aurora-A.

We next studied whether the presence of TPX2^1-43^ affected the binding of TACC3act to Aurora-A. We added unlabeled TPX2^1-43^ to AurA-DN along with ^15^N-labeled TACC3act and collected a ^1^H-^15^N HSQC spectrum (Fig 2D). The presence of TPX2^1-43^ did not significantly affect the chemical shifts of residues in the N-terminal region of TACC3act, which remained at their values obtained in the TACC3act/AurA-DN mixture. This confirms that TPX2^1-43^ does not displace Aurora-A from TACC3act, consistent with our GST co-precipitation data (Fig 2A). We conclude that TPX2^1-43^ and TACC3act must have largely distinct binding sites on Aurora-A. Notably, the additional chemical shift changes in TACC3act upon addition of TPX2^1-43^ were restricted to the vicinity of S558, which is the principal site phosphorylated by Aurora-A (Fig 2C and S1D Fig). In addition, chemical shift perturbations observed for the side-chain amides of Q557 indicate their involvement in the structural changes in TACC3act associated with the addition of TPX2^1-43^.

The interaction with Aurora-A involves more residues in TACC3 in the presence of TPX2^1-43^, but does this enhance the affinity of their interaction? We tracked the chemical shift changes of TACC3act as Aurora-A was titrated in, both in the presence and absence of TPX2^1-43^ (Fig 2E). We calculated a K_d_ of 8.2 ± 0.8 μM in the absence of TPX2^1-43^. In the presence of TPX2^1-43^, the K_d_ decreased to 1.1 ± 1.2 μM (Fig 2F). The overall enhancement of binding between TACC3act and Aurora-A in the presence of TPX2^1-43^, and the observation of binding at the phosphorylation site of TACC3, are consistent with the known role of TPX2^1-43^ in increasing the catalytic activity of Aurora-A.

Taken together, structural and biophysical studies reveal two Aurora-A binding sites on TACC3act: the first between residues 520–542 that binds independently of TPX2, and a second region centered on the S558 phosphorylation site that has TPX2-dependent binding. To corroborate these findings, we aimed to identify point mutations in TACC3act that disrupt the interaction with Aurora-A.

### The side chain of F525 is a major determinant of Aurora-A binding and activation

**Fig 3:**
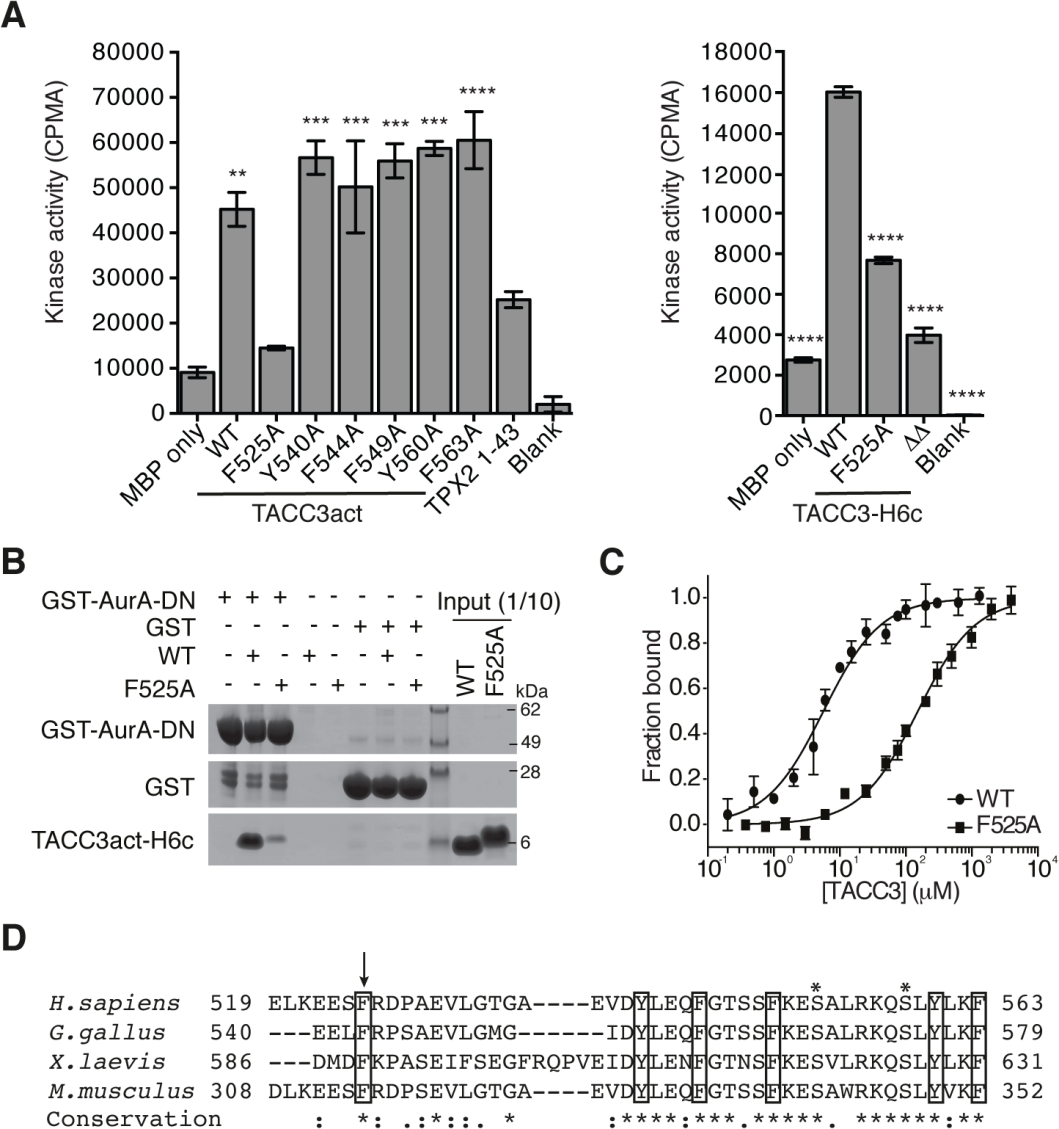
Mapping of key determinants for TACC3 binding to Aurora-A. (A) *In vitro* kinase activity assay of Aurora-A 122–403 in the presence of TACC3act, TACC3-H6c and TACC3 mutants. ΔΔ, TACC3-H6c Δ519–546 and Δ564–629. Stimulation of Aurora-A catalytic activity by TACC3 was determined by incorporation of ^32^P into MBP and quantified by scintillation counting. TPX2^1-43^ was used as a positive control for Aurora-A activation. Error bars represent the standard error for two independent reactions. ** = P<0.01, *** = P<0.001 and **** = P<0.0001 using one-way ANOVA with Dunnett's post-hoc test compared to the MBP only reaction (left) and compared to the WT reaction (right). (B) Co-precipitation assay to assess binding between GST-AurA-DN and TACC3act-H6c (WT) and the point mutant, TACC3act-H6c F525A (F525A). GST was used as a binding control. (C) Binding affinities of Aurora-A 122–403 C290A, C393A for TACC3act-H6c WT and F525A were determined using microscale thermophoresis. Data were fitted to Eq 2. Error bars represent the standard deviation of 3 measurements. (D) Multiple sequence alignment of TACC3 homologues within the minimal Aurora-A binding region. Asterisks above the alignment mark Aurora-A phosphorylation sites. Sequence conservation is shown below the alignment: ‘*’ indicates identical residues, ‘:’ identifies conservative substitutions and ‘.’ represents semi-conserved substitutions. Conserved aromatic residues are marked with black boxes. Residue F525 is marked with an arrow.

We performed alanine-scanning mutagenesis of the six conserved aromatic residues within TACC3act to identify the key binding residues. This strategy was based on the observation that aromatic residues form key interactions at the binding interface of protein-protein complexes, and are important in the function of intrinsically-disordered proteins, such as the Aurora-A binding region of TPX2 [48,51–53]. We found that F525 was the only aromatic amino acid within TACC3act that contributed to Aurora-A activation (Fig 3A). The F525A mutation also reduced the activation of Aurora-A by full-length TACC3, from 5-6-fold to 2-3-fold. Deletion of the entire region flanking the phosphorylation site (Δ519–546 and Δ564–629, TACC3ΔΔ) resulted in further reduction of Aurora-A activation. These findings suggest that residues additional to F525 within these deleted regions are involved in Aurora-A activation.

We then investigated whether the F525A mutation affected the binding affinity of TACC3 for Aurora-A. First, using GST co-precipitation assays, we found that TACC3act carrying the F525A mutation exhibited markedly reduced binding relative to wild-type (WT) TACC3act (Fig 3B). Since residual binding was detectable, we next quantified the affinity of the interaction. We established an assay to quantify the interactions of Aurora-A with binding partners using microscale thermophoresis (MST). We used a mutant version of the Aurora-A catalytic domain that has both surface-exposed cysteine residues (C290, C393) mutated to alanine [54]. These mutations stabilize the protein in solution, but do not significantly affect catalytic activity or structure [54,55]. The K_d_ of the interaction with WT TACC3act was determined to be 5.7 ± 0.4 μM (Fig 3C), a similar value to that determined using NMR spectroscopy, whereas the interaction with the F525A mutant had a K_d_ of 151.8 ± 9.3 μM (Fig 3C). We conclude that F525 makes an important contribution to the binding and activation of Aurora-A by TACC3.

The human TACC3 residues implicated in complex formation with Aurora-A are well-conserved in mouse, chicken and *Xenopus* homologues (Fig 3D). All four TACC3 homologues have a phenylalanine residue at the position equivalent to the human F525. Indeed, this phenlyalanine residue is essential for binding of full-length *Xenopus* TACC3 (maskin) to Aurora-A (S3A Fig). All four also have a basic residue at the position equivalent to R526 and a proline residue nearby, although the position varies across the four organisms, and is either immediately C-terminal to the basic residue or one residue further along.

### Aurora-A activation and phosphorylation functions of TACC3 can be dissected by point mutations

**Fig 4:**
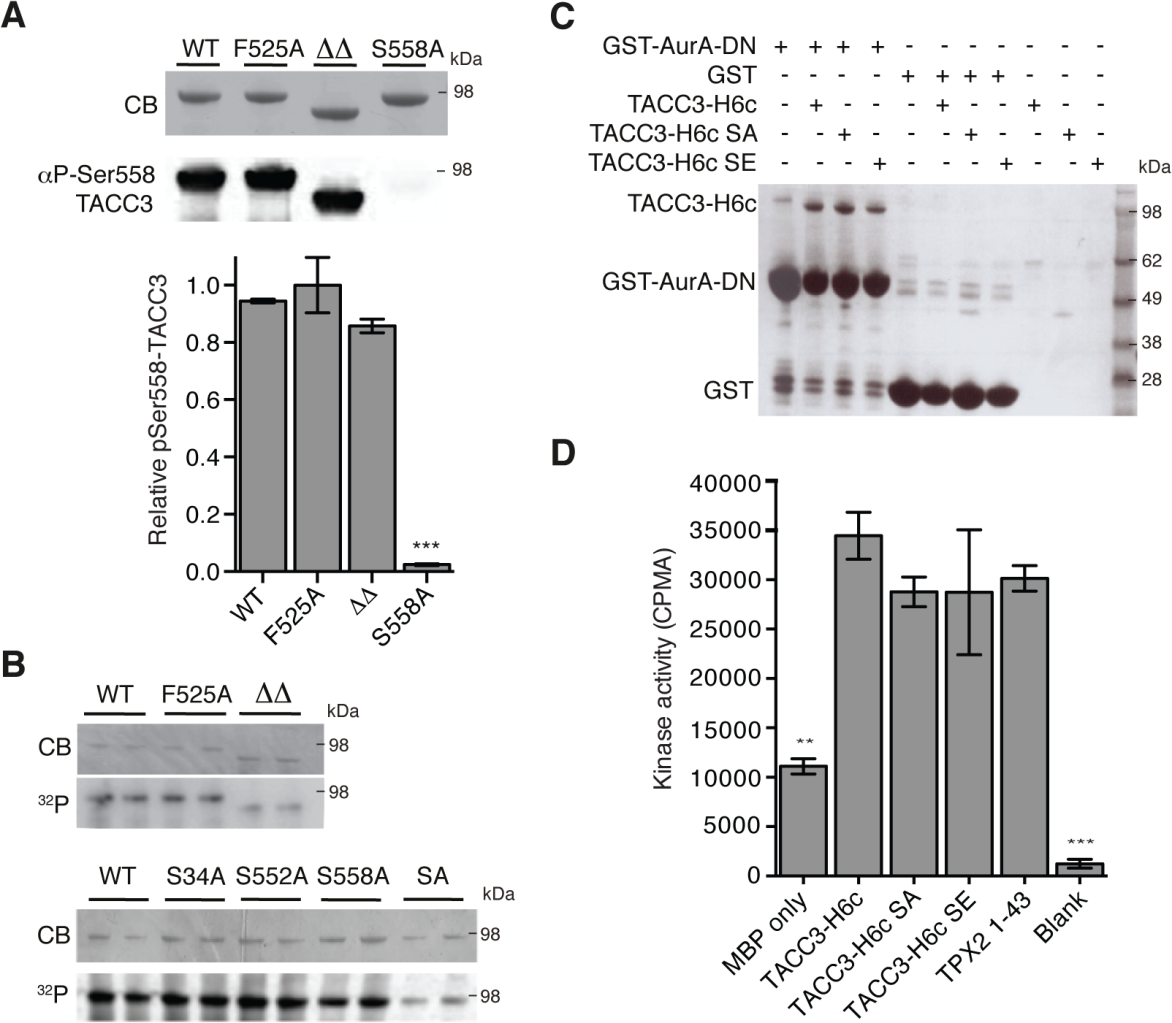
Biochemical characterisation of TACC3 mutants defective in either Aurora-A phosphorylation or activation. (A) Ser558 phosphorylation of TACC3-H6c WT, F525A, ΔΔ (TACC3-H6c Δ519–546 and Δ564–629) and S558A by Aurora-A was measured by quantitative immunofluorescent blotting using an antibody specific to phosphorylated Ser558 in TACC3 (middle, the scanned blot was converted to black and white). The coomassie-stained gel is shown above. Quantification of phosphorylation is shown below. Error bars represent the standard error for two independent reactions. *** = P<0.001 using one-way ANOVA with Dunnett's post-hoc test compared to the WT reaction. (B) Total phosphorylation of TACC3-H6c WT, ΔΔ and point mutants by Aurora-A was monitored by incorporation of ^32^P-labelled ATP (bottom). Phospho-null (SA) has three mutations: S34A, S552A, S558A. Coomassie-stained gels are shown above. (C) Co-precipitation assay to assess binding between GST-AurA-DN and TACC3. The assay used wild-type, phospho-null (SA) and phospho-mimic (SE) TACC3-H6c as prey proteins. GST was used as a binding control. (D) Activation of Aurora-A by TACC3-H6c WT, SA and SE was monitored by *in vitro* kinase activity assay. The catalytic activity of Aurora-A was determined by incorporation of ^32^P into MBP and quantified by scintillation counting. TPX2^1-43^ was used as a positive control for Aurora-A activation. Error bars represent the standard error for two independent reactions. ** = P<0.01 and *** = P<0.001 using one-way ANOVA with Dunnett's post-hoc test compared to the WT reaction.

The region of TACC3 responsible for mediating Aurora-A activation is distinct from, albeit close to, the phosphorylation site at S558, raising the question of whether these two functions are independent. We investigated whether mutants of TACC3 that were deficient in Aurora-A activation were efficient substrates. Recombinant full-length TACC3 WT, F525A and ΔΔ were incubated with Aurora-A kinase and the extent of phosphorylation was determined by quantitative immunofluorescence using a anti-phospho-S558 TACC3 antibody (Fig 4A) and autoradiography to measure ^32^P-label incorporation (Fig 4B). All TACC3 protein variants were equally well phosphorylated on Ser-558 but total phosphorylation of TACC3ΔΔ was reduced compared to TACC3 WT and F525A. Therefore, at least *in vitro*, the activation of Aurora-A by TACC3 can be disrupted by the F525A mutation, whilst maintaining phosphorylation of TACC3.

To address whether the phosphorylation state of TACC3 influenced Aurora-A binding and activation, we generated TACC3 variants in which all three Aurora-A phosphorylation sites (S34, S552 and S558) were mutated to either alanine (SA, phospho-null mutant) or glutamic acid (SE, phospho-mimic mutant). The SA mutant had strongly reduced levels of phosphorylation compared to the individual mutations. (Fig 4B). Binding of TACC3-H6c to GST-Aurora-A was assayed by GST co-precipitation assay using GST as a control (Fig 4C). We observed specific interaction of all three TACC3 variants to the Aurora-A catalytic domain. TACC3 phospho-null and phospho-mimic mutants enhanced Aurora-A kinase activity by a similar amount, using an *in vitro* kinase assay (Fig 4D). Similar results were obtained using recombinant *Xenopus* proteins (S3B and S3C Fig).

In summary, biochemical and structural studies have identified a sub-region of the TACC3 CID centered on F525 that binds Aurora-A and stimulates its kinase activity, independent of the phosphorylation state of S558. This raises the question of what might be the function of the TACC3 dependent Aurora-A activation during mitosis since it is well established that Aurora-A is efficiently activated by TPX2.

### The F543 residue is required for efficient accumulation of TACC3 on the mitotic spindle

**Fig 5:**
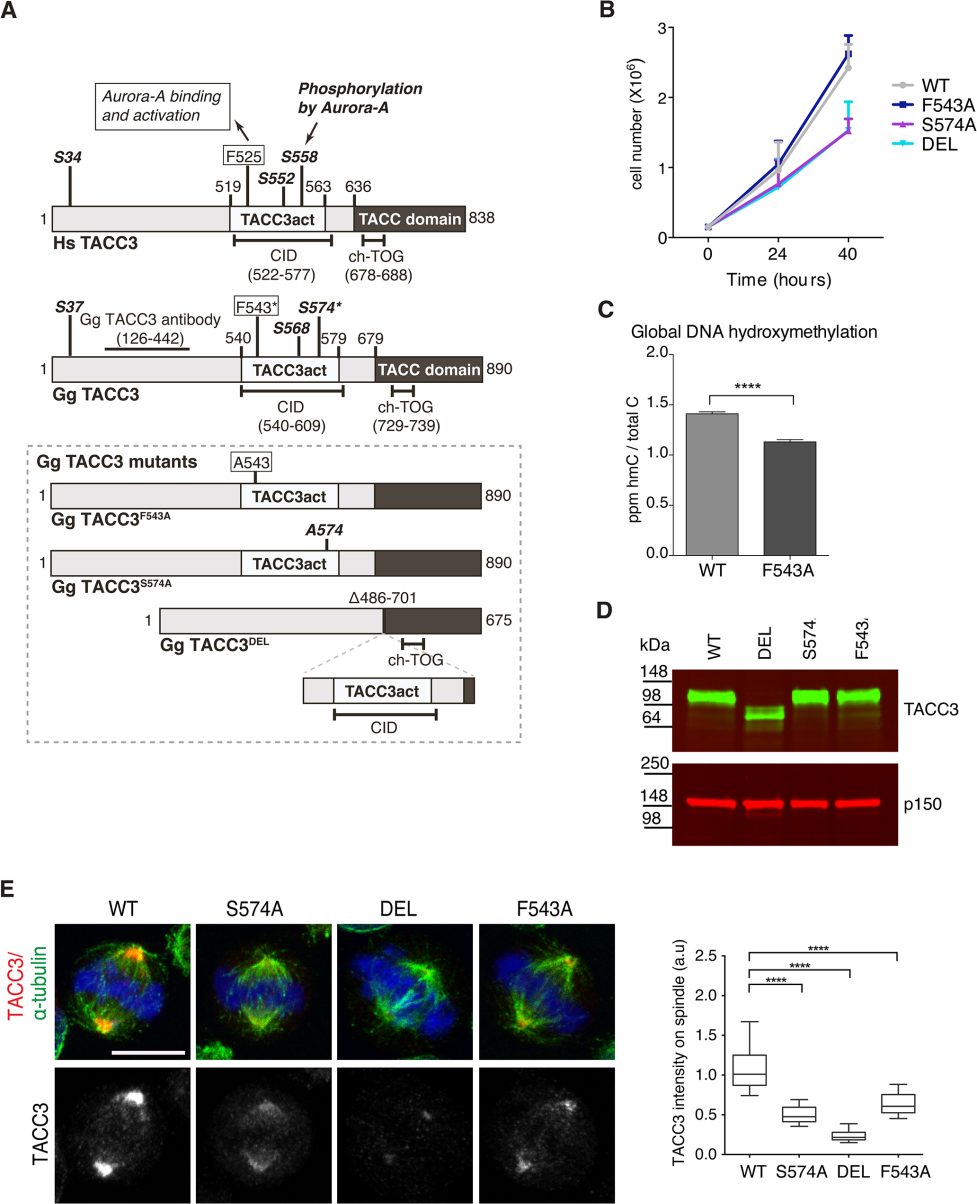
F543 in TACC3 is required for efficient targeting of TACC3 to the mitotic spindle. (A) Graphical illustration of domain organization, numbering and properties of key residues in *Homo sapiens (Hs)* and *Gallus gallus (Gg)* TACC3 proteins. Framed area below shows properties of mutant TACC3 protein products expressed in F543A, S574A and DEL cells, respectively. (B) Growth curves are shown for WT and mutant cell lines. n = 3 technical replicates, error bars represent standard deviation. (C) Measurement of 5-hydroxymethylcytosine (hmC) by tandem liquid–chromatography mass spectrometry in cells. hmC levels are expressed as parts per million (ppm) of total cytosines or ‘C’. Note that hmC levels inversely correlate with proliferation rate [56]. n = 3 technical replicates. Statistical significance was assessed using t-test (**** P < 0.0001). (D) Western blot shows TACC3 protein levels in the DT40 cell lines with genotypes as indicated. The p150 subunit of dynactin serves as loading control. (E) TACC3 localisation to the mitotic spindle is impaired in all three mutant DT40 cell lines. In merged images α-tubulin is green, TACC3 is red and DNA is blue. Scale bar = 5 μm. Box plot on right depicts intensity of TACC3 staining on the mitotic spindle. TACC3 signal intensity was quantified in mitotic spindle volumes defined by α-tubulin staining. A minimum of 60 cells was scored per genotype. Statistical significance was assessed using t-test (**** P < 0.0001).

To test the respective contributions to TACC3 function by the F525 and S558 residues, we have introduced defined mutations in the *Tacc3* gene using homologous gene targeting in the chicken B cell line, DT40 (S4–S6 Figs). Fig 5A depicts domain organization and numbering of key residues in human and chicken TACC3. Homozygous mutant cell lines were generated in which either F543 or S574 (equivalent to F525 and S558 in human TACC3, respectively) were replaced with alanine (referred to as F543A or S574A modifications). A further cell line was created in which the CID of TACC3 was removed through deletion of exons 5–9 encoding amino acids 486–701 (referred to as DEL modification). It is worth noting that the ch-TOG interaction domain is intact in the TACC3 mutants (Fig 5A)[36]. In all three cases, sequential gene targeting was employed to edit both alleles of *Tacc3*. We have obtained multiple independently derived homozygous clones of DEL, S574A and F543A cells, all of which were morphologically normal and viable. F543A was similar to WT, whereas DEL and S574A cells grew more slowly (Fig 5B). To further evaluate proliferation rates in WT and F543A cells, we turned to a recently developed mass-spectrometry-based method that measures levels of 5-Hydroxymethylcytosine (hmC) in DNA; previous studies revealed an inverse correlation between hmC levels and cell proliferation [56]. Remarkably, the F543A cells exhibited reduced hmC, indicative of faster proliferation (Fig 5C). Western blots revealed comparable TACC3 expression levels in WT, S574A and F543A cells, and a reduction of ~50% in DEL cells (Fig 5D). The point mutant TACC3 products, TACC3^S574A^ and TACC3^F543A^, and WT TACC3, ran at ~100kDa, whereas DEL cells expressed a shorter product of ~75kDa, termed TACC3^DEL^.

Subcellular localisation of TACC3 in DT40 cells is similar to what has been described in other model systems; TACC3 is centrosomal in G2, and from nuclear envelope breakdown (NEBD) onwards it accumulates at the mitotic spindle poles and decorates spindle MTs (Fig 5E and S7A and S7B Fig). In human cells TACC3 localisation to the mitotic spindle is markedly enhanced by phosphorylation of S558, most likely because this modification promotes interaction with clathrin, which in turn generates a composite MT-binding interface between TACC3 and clathrin [36]. Indeed, TACC3^DEL^ that lacks the CID, including the S558-equivalent Aurora-A phosphorylation site, exhibited a very weak signal restricted to spindle poles (Fig 5E). Although much reduced compared to wild-type TACC3, TACC3^S574A^ and TACC3^F543A^ were readily detectable on the mitotic spindle. Quantification of TACC3 signal intensities on mitotic spindles confirmed these findings (Fig 5E). As in WT cells, TACC3 was detectable on centrosomes in G2 and prophase S574A, DEL and F543A cells, but at much reduced levels (S7A Fig). In human cells TACC3 is essential for localising ch-TOG to the mitotic spindle but not to centrosomes [57]. Consistently, in S574A, F543A and DEL cells ch-TOG levels followed TACC3 levels on the spindle, with little change in centrosomal amounts (S7C and S7D Fig). To test the MT-binding capacity of the mutant TACC3 proteins, MT-pelleting assays were performed using paclitaxel-stabilised MTs from cell extracts. TACC3^S574A^, TACC3^F543A^, and even TACC3^DEL^ that lacks the CID, all co-sedimented with MT polymers, although less efficiently than wild-type TACC3 (S7E Fig). Thus, significant amounts of TACC3 can interact with MTs independently of CID at least *in vitro*, most likely through the TACC domain.

In summary, we find that F543 is required for normal mitotic spindle localization of TACC3, similar to S574, prompting us to investigate the mechanism underlying reduced binding of TACC3^F543A^ to spindles.

### The F543 residue increases levels of phosphorylated TACC3 and promotes interaction between TACC3 and clathrin

**Fig 6:**
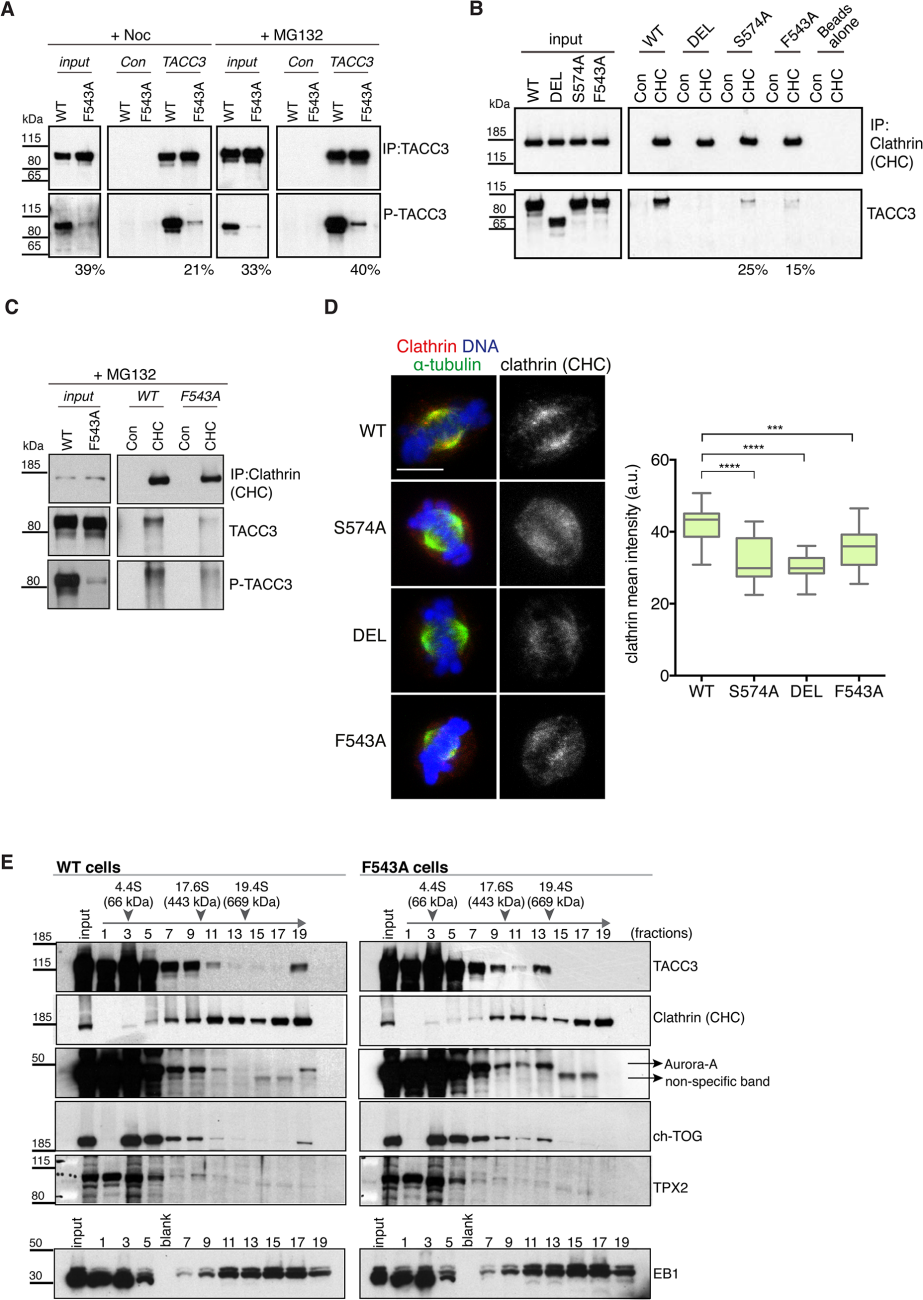
The F543A mutation impairs phosphorylation of TACC3 and its interaction with clathrin. (A) Western blots show immunoprecipitation of TACC3 from DT40 cell extracts. Cells were blocked in mitosis by nocodazole (+Noc) for 16 hours or released from nocodazole block into MG132 for 1 hour (+MG132). Genotypes are as indicated. Antibodies against TACC3 or random rabbit IgG (Con) were used for immunoprecipitation (IP). Inputs represent cytoplasmic extracts. Blots were probed with anti-TACC3 or anti-phospho-S574-TACC3 (P-TACC3) antibodies. The percentages below the plots refer to P-TACC3 signal levels in the mutants relative to WT. Band intensities were analysed on films with ImageJ; P-TACC3 signal was normalized against total TACC3 (inputs) or total immunoprecipitated TACC3 for each genotype. (B) Western blots show co-immunoprecipitation of clathrin and TACC3 from DT40 cell extracts. Genotypes are as indicated. Antibodies against clathrin heavy chain (CHC) or random rabbit IgG (con) were used for immunoprecipitation (IP). Inputs represent cytoplasmic extracts. Blots were probed with anti-CHC or anti-TACC3 antibodies, as indicated. The percentages below the plots refer to TACC3 signal levels in F543A relative to WT. Band intensities were analysed on films with ImageJ and TACC3 signal was normalized against total immunoprecipitated clathrin for each genotype. (C) Western blots show co-immunoprecipitation of clathrin with P-TACC3 from WT and F543A DT40 cell extracts. Antibodies against clathrin heavy chain (CHC) or random rabbit IgG (con) were used for immunoprecipitation (IP). Inputs represent cytoplasmic extracts. Blots were probed with anti-CHC, anti-TACC3 or anti P-TACC3 antibodies, as indicated. (D) Localisation of clathrin in mitotic cells is shown on left. In merged images α-tubulin is green, clathrin (CHC) is red and DNA is blue. Scale bar = 5 μm. Box plot on right depicts mean intensity of clathrin staining on the mitotic spindle. Signal intensity of clathrin (CHC) antibody was quantified in mitotic spindle volumes defined by α-tubulin-FITC staining. A minimum of 25 cells was scored per genotype. Statistical significance was assessed using t-test (***P = 0.0001, **** P < 0.0001). (E) Western blots of sucrose gradient centrifugation from WT and F543A cytoplasmic cell extracts. Note different fractionation patterns between genotypes: in F543A cells fraction 13 contains increased amounts of ch-TOG, TACC3 and Aurora-A, but not clathrin. TPX2 and EB1 display similar fractionation patterns between WT and F543A cells. Position of molecular weight markers (BSA: 4.4S; Apoferritin: 17.6S; Thyroglobulin: 19.4S) on the gradient is shown.

TACC3 is found in a complex with ch-TOG and clathrin [32,33,35]. Recent studies suggest that clathrin and TACC3 are mutually dependent for efficient spindle localization, and binding between clathrin and TACC3 requires phosphorylation at S558 by Aurora-A [31–33,35]. Therefore, we next asked if reduced spindle localization of TACC3^F543A^ was due to impaired phosphorylation and/or clathrin binding. S588/S574 phosphorylation status was assayed using a phospho (P)-TACC3 antibody raised against P-S558. This antibody is specific, since no signal is detected when TACC3 is immunoprecipitated from S574A and DEL cells (S8A Fig). Like WT, F543A cells contained P-TACC3 albeit at reduced levels. Because immunoprecipitation could introduce a bias, we wanted to further examine the phosphorylation status of TACC3^F543A^ in cytoplasmic cell lysates. By increasing the concentration of lysates to over 5 mg/ml, we were able to detect P-TACC3 in nocodazole-blocked mitotic WT cells and observed a major reduction in P-TACC3^F543A^ levels in F543A cells (Fig 6A). Reduction in P-TACC3^F543A^ was sustained after releasing cells from nocodazole block into the proteasome inhibitor, MG132, which leads to accumulation of metaphase cells. These findings indicate that the F543A mutations negatively impacts on phosphorylation at S574 both in prometaphase and metaphase. In immunofluorescence analysis, the P-TACC3 antibody exhibited weak spindle pole staining in F543A cells (S8B Fig). Since TACC3^F543A^ showed reduced phosphorylation, we next asked if the F543A mutation also affected the binding and localization of clathrin. Immunoprecipitations of clathrin revealed reduced TACC3 binding both in S574A and F543A cells (Fig 6B). A complete loss of interaction was seen in DEL cells, which lack the CID. Despite the major reduction in P-TACC3 levels in F543A cells, clathrin co-immunoprecipitated with P-TACC3^F543A^, indicative of intact P-TACC3-clathrin complexes in these cells (Fig 6C). Consistent with these findings, we noted a significant decrease in spindle localization of clathrin in all three mutant lines, but the effect was again greater in S574A and DEL than in F543A cells (Fig 6D). However, even in DEL cells, detectable levels of clathrin remained on the spindle, confirming results from human cells that at least some clathrin can localize to the spindle independently of TACC3 [31–33].

To further interrogate the effect of the F543A mutation on TACC3 protein complexes, we performed sucrose gradient sedimentation using cytoplasmic extracts of WT and F543A cells (Fig 6E). When compared to WT, in F543A cells we noted an accumulation of TACC3, ch-TOG and Aurora-A, but not clathrin, in fraction 13 that corresponds to protein complexes of ~400–600 kDa. The actual stoichiometry of the individual components is not known, but since TACC3 is likely to form a dimer, we estimate the size of TACC3-ch-TOG-Aurora-A complex to fall within the range of ~350–500 kDa. By contrast, the molecular weight of a complex containing clathrin would be nearer to 1 MDa, if clathrin indeed formed a triskelion in this context. These results suggest that the F543A mutation could instigate a shift towards a TACC3-ch-TOG-Aurora-A complex away from the clathrin-TACC3-ch-TOG-Aurora-A MT-crosslinking complex.

### TACC3 is dispensable for global activation of Aurora-A in cells

Our *in vitro* data suggested that the F543A point mutation significantly reduced the ability of TACC3 to activate and bind Aurora-A, prompting us to investigate whether TACC3 also plays a key role in activating Aurora-A kinase *in vivo*. We demonstrated reduced phosphorylation and clathrin-binding of TACC3^F543A^ (Fig 6), and both these phenotypes could result from a decrease in global Aurora-A activity. Aurora-A kinase is autophosphorylated at T288 of the T loop and this phosphorylation event is widely considered as a surrogate marker for kinase activity [45,46]. Thus, we tested the effects of RNA interference-mediated depletion of TACC3 on T288 phosphorylation of Aurora-A in Jurkat lymphocytes. We observed no correlation between TACC3 levels and the extent of T288 autophosphorylation in these cells (S8C Fig). Specificity of antibodies was confirmed by treatment of cells with the ATP-competitive Aurora-A inhibitor, MLN8054 [7]. Similar stainings could not be performed in DT40 cell lines due to lack of antibodies specific to chicken phospho-Aurora-A and our multiple attempts to generate such antibodies also failed. However, we have recently shown that Aurora-A in which T288 has been mutated to alanine still exhibits activity when bound by TPX2, suggesting that T288 phosphorylation may not be the best surrogate marker for Aurora-A catalytic activity [49]. To assay Aurora-A activity independently of its phosphorylation status, the kinase was immunoprecipitated from extracts of WT, DEL and F543A DT40 cells (S8D Fig). We found no difference between the cell lines in the levels of phosphorylation of a surrogate substrate, MBP. Moreover, Aurora-A localization to the spindle, a process known to depend on TPX2 and Aurora-A kinase activity, was normal in the TACC3 mutant cells (S8E Fig) [10,15,58]. However, in agreement with our *in vitro* data on human TACC3, co-immunoprecipitation experiments showed reduced binding between TACC3^F543A^ and Aurora-A (S8F Fig). Taken together, TACC3 is not a major regulator of global Aurora-A activity, but this does not exclude a more local role for the Aurora-A-TACC3 interaction.

### The F543A mutation accelerates mitosis while maintaining faithful chromosome segregation

**Fig 7:**
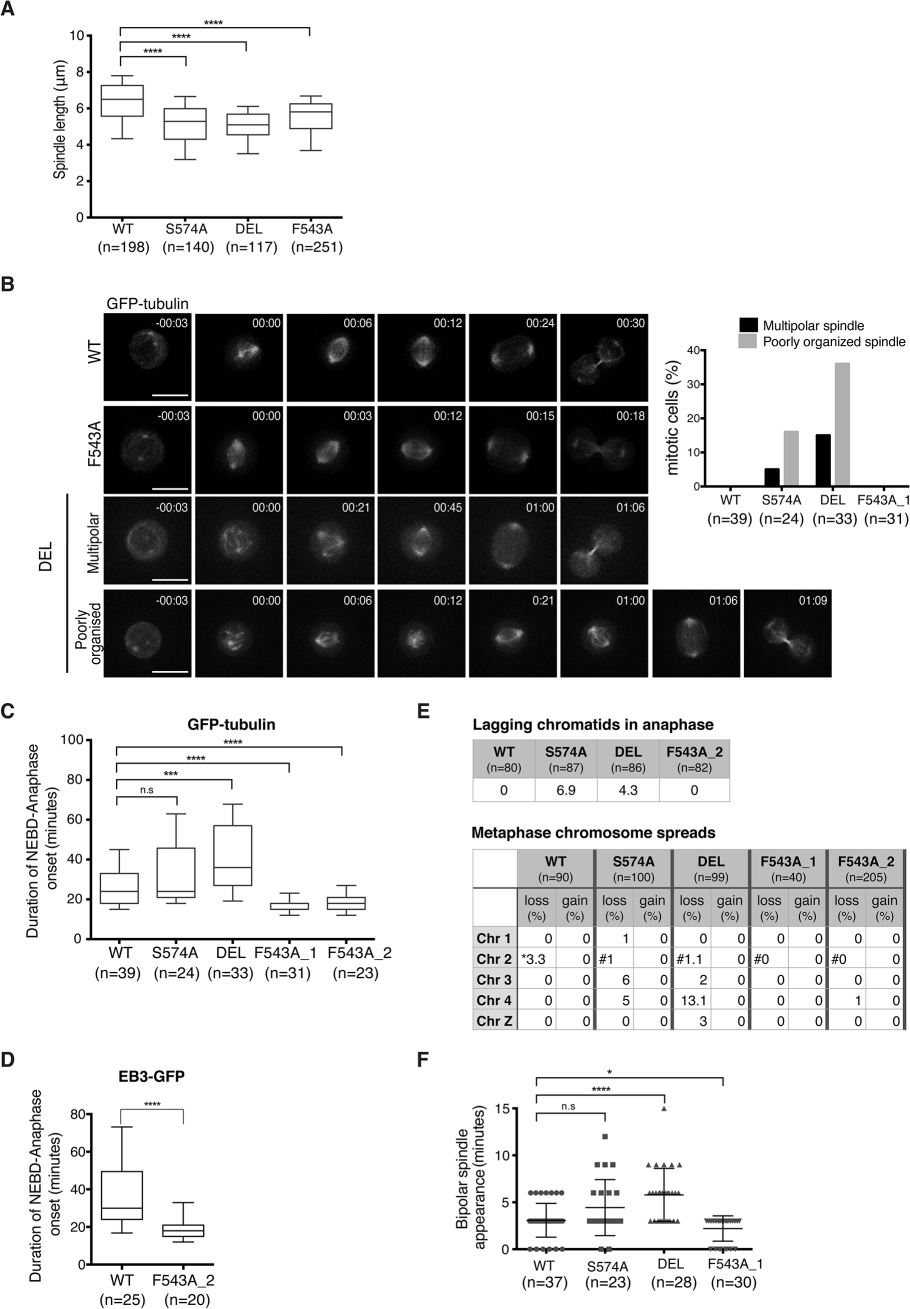
Spatially distinct residues in TACC3 dictate mitotic duration and fidelity. (A) Spindle length measured in 3D using Volocity. Number of cells analysed is indicated in graph (n). Student t-test (**** P < 0.0001). (B) Mitotic spindle morphologies (as described in main text) observed during time-lapse microscopy of GFP-tubulin-expressing TACC3 mutant cell lines. Representative still frames are shown on left. Scale bar = 5 μm. (C) Durations of NEBD to anaphase onset obtained from time-lapse microscopy performed on GFP-tubulin-expressing TACC3 mutant cell lines. F543A_1 and F543A_2 cells represent independently derived clones. Note that we observed no correlation between GFP levels and mitotic timing, or between mitotic timing and time of NEBD with respect to duration of filming in these experiments. Box plot shows 10–90 percentiles for each genotype. Number of cells analysed is indicated in graph (n). Mann Whitney nonparametric t-test (**** P < 0.0001, *** P < 0.001 and n.s. stands for ‘no significance’). (D) Durations of NEBD to anaphase onset obtained from time-lapse microscopy performed on EB3-GFP-expressing cells. Box plot shows 10–90 percentiles. Number of cells analysed is indicated in the graph (n). Mann Whitney nonparametric t-test. (**** P < 0.0001). (E) Analysis of chromosome segregation. Top table shows percentage of lagging chromatids seen during anaphase in fixed cells. Bottom table shows results from metaphase chromosome (chr) spreads. Number of autosomes 1, 2, 3, 4 and the sex chromosome, Z was analysed in cells. Frequencies of loss or gain of a single copy of individual chromosomes are indicated. Note that WT DT40 cells are trisomic for chromosome 2, whilst all the TACC3 mutants are diploid. Therefore, cells with two or three copies of chromosome 2’s are marked with ‘*’ or ‘#’, respectively. (F) Plot depicts time between NEBD and bipolar spindle appearance in time-lapse microscopy from Fig 7B. Cells with multipolar spindles and cells in which one of the two spindle poles was outside of the Z stack range for these time points were excluded from analysis. Number of cells analysed is indicated in graph (n). Mann Whitney nonparametric t-test (* P = 0.04, **** P < 0.0001).

To assess the functional consequences of the F543A mutation in TACC3, we examined mitotic spindle morphology in the mutants. TACC3 depletion in human cell lines causes shorter mitotic spindles, a phenotype observed in all three mutant DT40 cell lines (Fig 7A) [57]. Spindles were shorter in DEL and S574A than in F543A cells, consistent with more TACC3 being retained on spindle poles in the latter. Since all three mutants show reduced S574 phosphorylation and clathrin binding, Aurora-A-dependent formation of the TACC3-clathrin-ch-TOG MT crosslinking complex could be important for normal spindle length. Alternatively, TACC3 may promote MT stability through associating with ch-TOG, and phosphorylation in this case could help targeting of TACC3 onto the mitotic spindle.

Although mitotic spindles were largely intact and bipolar in fixed cells, time-lapse microscopy of DT40 cells expressing GFP-α-tubulin revealed transient abnormal spindle organisation both in S574A and DEL cells (Fig 7B). The category ‘poorly organized spindle' describes mitotic cells that contain a mitotic spindle and additional cytoplasmic MT asters, the latter lasting for 1–2 frames (36% in DEL and 16% in S574A). The category ‘multipolar spindle' depicts cells with extra spindle poles that are sustained for a minimum of 3 frames (15% in DEL and 5% in S574A) (Fig 7B). Despite these abnormal structures, only a single DEL cell out of 33 exhibited multipolar cell division. Upon disruption of TACC3 function, a mitotic delay has been observed in several model systems [33,57,59]. Indeed, a marked delay in NEBD-anaphase duration was seen in DEL cells and a slight delay was also detected in S574A cells, but this failed to reach statistical significance (Fig 7C). By contrast, F543A cells exhibited significantly accelerated mitosis, a phenotype confirmed in two distinct F543A cell clones. Similar results were obtained in EB3-GFP-expressing F543A cells (Fig 7D).

Reduced mitotic duration can be a consequence of sub-optimal functioning of the SAC, and in human cells this is observed upon loss of activity of the SAC components, Mad2 and BubR1 [13,60]. However, multiple lines of evidence suggest that fast mitosis in F543A cells is not caused by a failure of SAC. First, BubR1 accumulates at kinetochores similarly between F543A and WT cells (S9A Fig), and second, F543A cells arrest in mitosis upon treatment with low doses of nocodazole and paclitaxel (S9B Fig). Third, unlike DEL and S574A cells, F543A cells showed no lagging chromatids in anaphase and minimal aneuploidy based on the number of autosomes 1–4 and sex chromosome Z in metaphase spreads (Fig 7E). The most common source for lagging chromatids is merotelic kinetochore attachments, and our results therefore expose a role for Aurora-A-dependent phosphorylation of TACC3 in reducing merotely and thus chromosome missegregation. TACC3 has been reported to localize to the nuclear envelope, and thus we wondered if the nuclear envelope and its cell cycle-dependent regulation are intact in the mutants [61]. However, we found no evidence of impaired coordination of NEBD and chromosome condensation in these cell lines (S9C Fig). Another way to ‘speed up’ mitosis is via regulation of centrosome separation. Centrosome separation that is completed before NEBD shortens the time between NEBD and anaphase onset, accelerates bipolar spindle formation and increases the fidelity of chromosome segregation in HeLa cells [62–64]. Nevertheless, time-lapse imaging revealed no evidence for a function of TACC3 in centrosome separation (S9D Fig). While analyzing these time-lapse experiments, we however noted a difference in the timing of appearance of bipolar spindles in the TACC3 mutant cell lines. It occurred particularly early in F543A cells, only 2.2 ± 1.3 minutes after NEBD, compared to 3 ± 1.8 minutes in WT and 5.7 ± 2.8 minutes in DEL cells (Fig 7F). Fast bipolar spindle assembly in F543 cells could in part account for the early anaphase onset.

### TACC3 mutant proteins are efficiently recruited to nascent MT foci, but the appearance of bipolar spindles is delayed in S574A and DEL cells

**Fig 8:**
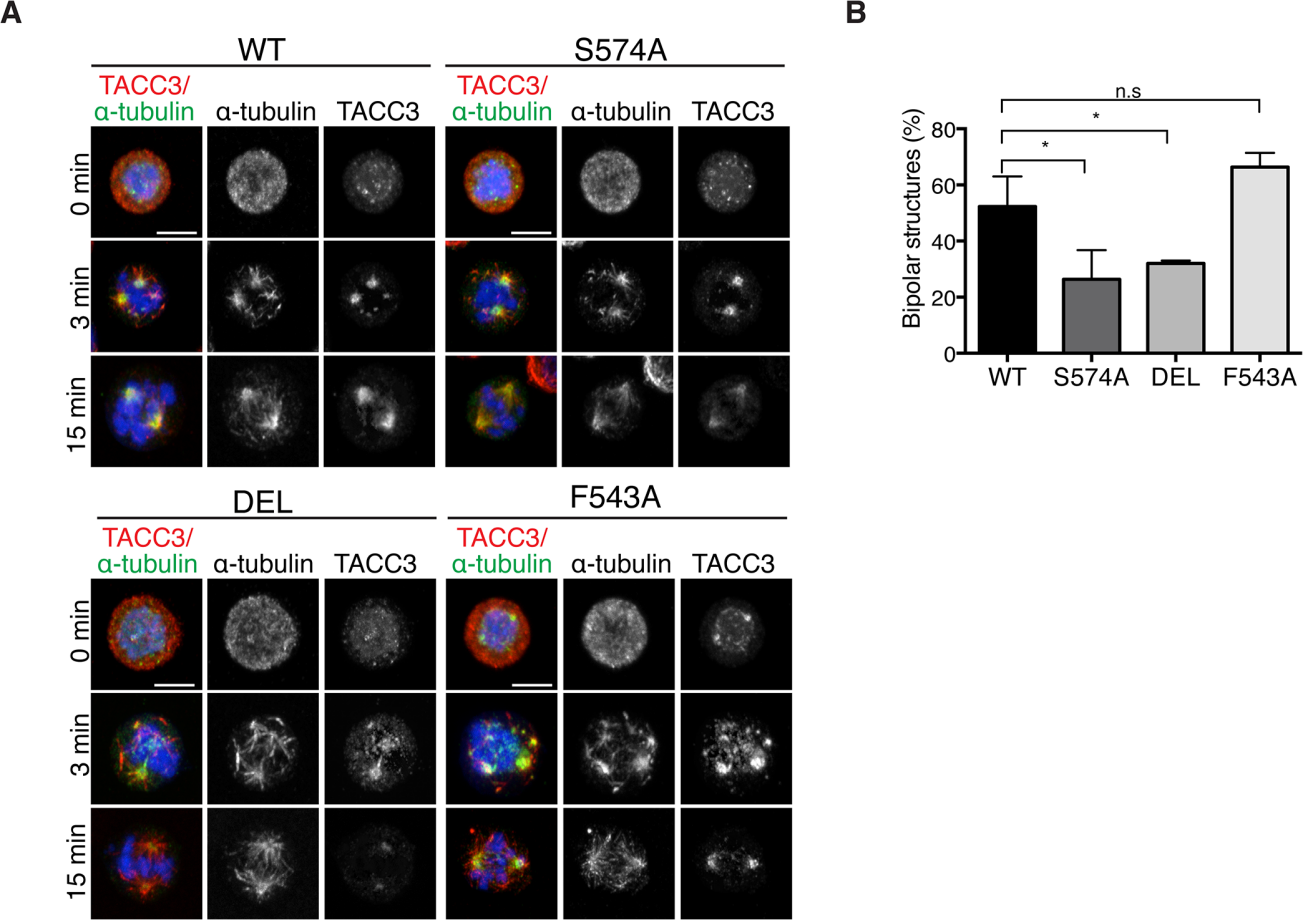
The appearance of bipolar spindles is delayed in S574A and DEL cells following MT regrowth. (A) MT regrowth assays in DT40 cells with genotypes as indicated. MTs were depolymerized by nocodazole and then allowed to recover for 3 or 15 minutes. Note that the mutant TACC3 products accumulate on nascent MTs. Scale bar = 5 μm. (B) Graph depicts percentages of mitotic cells with discernible bipolar spindles after 15 minutes of MT regrowth. n = 3 independent experiments; minimum of 100 cells scored per genotype per experiment. Student t-test (* P = 0.03).

Mitotic spindle assembly relies on the rapid formation of MTs generated primarily by centrosome- and chromatin-dependent pathways and to a lesser extent by spindle MT-driven MT nucleation followed by their subsequent organization into a bipolar array [1]. Cooperation of the MT assembly pathways considerably speeds up bipolar spindle assembly and kinetochore capture [1,4]. In addition to its role in centrosomal MT assembly, TACC3 has been recently described as a regulator of acentrosomal chromatin-dependent MT formation and kinetochore capture [22]. To assess if TACC3 could bind chromatin-associated MTs in DT40 cells, MTs were depolymerized and regrowth assays were performed. 3 minutes after initiating regrowth, centrosomal MT asters and chromatin-associated MT foci became apparent in WT and mutant DT40 cells (Fig 8A). The sizes of these asters were highly variable and thus we could not decipher genotype-specific changes. By 15 minutes however, unlike DEL and S574A cells that seemed to lag behind WT cells in achieving spindle bipolarity, F543A cells could form robust bipolar spindles as efficiently as WT (Fig 8B). In line with our finding that the mutant TACC3 products co-pelleted with MTs *in vitro* (S7E Fig), TACC3^S574A^, TACC3^F543A^ and to a lesser extent, TACC3^DEL^, all accumulated on nascent centrosomal and chromatin-associated MT foci at the 3-minute time point. However, by 15 minutes after regrowth, they adopted their respective patterns as seen in untreated mitotic cells (Fig 8A). This suggests that TACC3 associates with centrosomal and chromatin-nucleated MTs independent of Aurora-A phosphorylation and clathrin binding, but its retention on MTs requires these factors.

## Discussion

**Fig 9:**
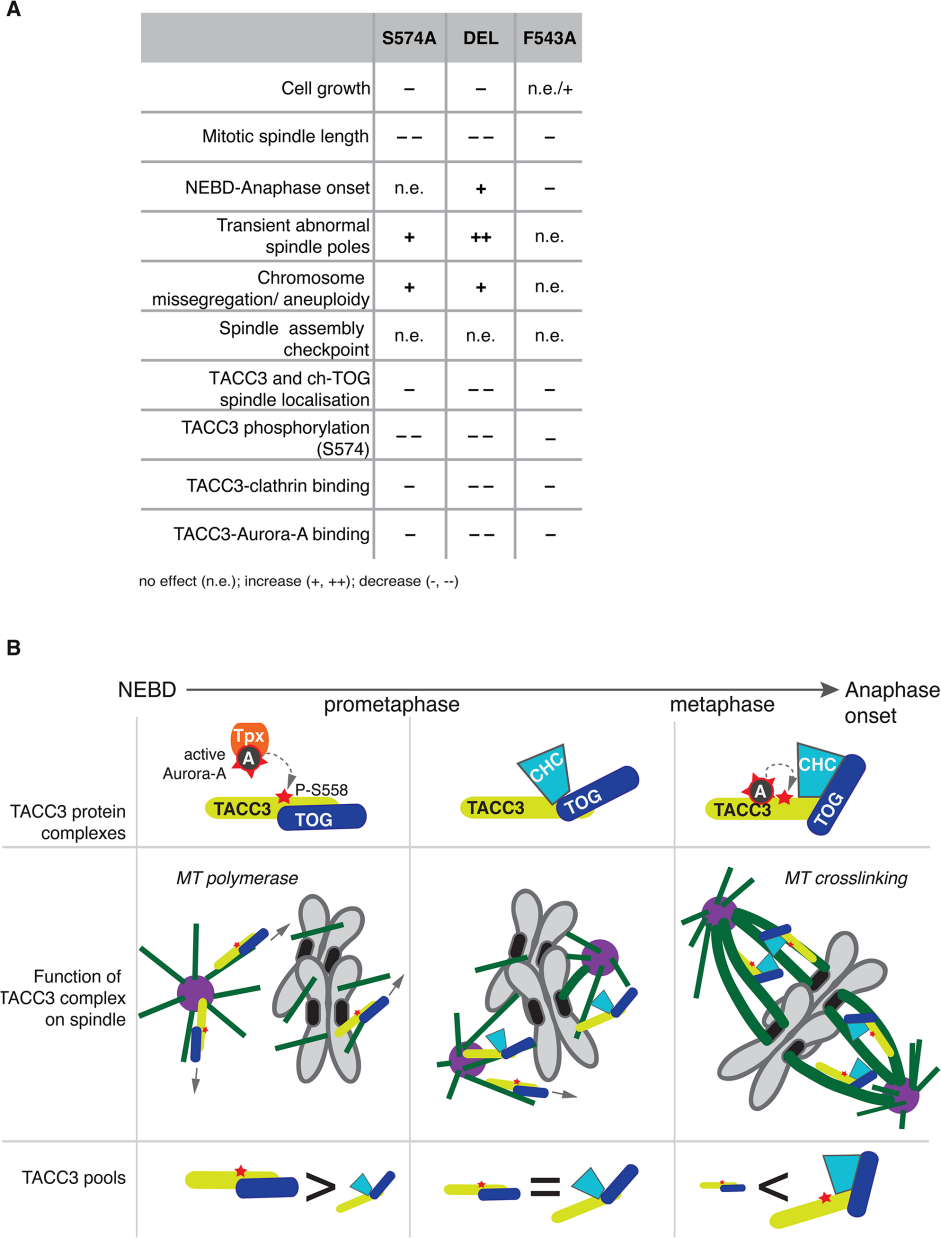
Summary and working model. (A) Table summarises phenotypes observed in the DT40 TACC3 mutants. Cell doubling time of F543A is similar to WT, but hmC levels indicate faster proliferation, hence the n.e./+ description. (B) Model illustrates roles of different TACC3 pools during mitosis. Briefly, in prometaphase TACC3 is phosphorylated on S558 by TPX2-bound Aurora-A, and P-TACC3 enhances MT polymerase activity of ch-TOG both near centrosomes and chromatin. As kinetochore MTs become established, TACC3 recruits clathrin to these MTs, but the complex is initially unstable. Long-lived kinetochore MTs allow local activation of Aurora-A by TACC3 and phosphorylation at S558 stabilises the TACC3-clathrin complex, which in turn crosslinks these MTs.

This study describes the long-term cellular effects of TACC3 mutations that preclude or substantially reduce Aurora-A-dependent phosphorylation and clathrin binding. Although both the phosphorylation at S574 and the interaction between clathrin and TACC3 are perturbed in S574A and F543A cells, the mutations have distinct effects on several aspects of mitosis (see Fig 9A for summary of phenotypes). Unlike S574A cells that show slightly impaired mitotic spindles, an increase in aneuploidy and a minor delay in mitosis, F543A cells display robust spindle formation and increased mitotic speed. Thus, in addition to its role in mediating clathrin-TACC3 complex formation, the interaction between TACC3 and Aurora-A seems important for the temporal control of spindle assembly.

### TACC3 has clathrin-dependent and–independent roles during mitosis

The role of TACC3 in cross-linking and stabilizing k-fibres in a complex with clathrin is now well established [32,33,35,36]. We have dissected the CID of TACC3, which consists of two distinct regions: a previously reported domain centered on the Aurora-A phosphorylation site at S558 and the di-leucine motif [36]; and a new Aurora-A binding region centered on F525. In particular, our *in vivo* studies suggest that the S558 phospho-residue and F525 site play comparable roles both in TACC3 localisation and clathrin binding. Intriguingly, however, the findings that TACC3^S574A^, TACC3^DEL^ and TACC3^F543A^ are defective in binding clathrin, yet i) co-sediment with MTs *in vitro*, ii) decorate nascent MTs during regrowth experiments and iii) associate with spindle MTs to varying degrees, collectively argue against an essential role for clathrin in the recruitment of TACC3 to spindle MTs (Fig 9A). This is consistent with reports that unlike depletion of TACC3, which abolishes clathrin recruitment to spindle MTs, depletion of clathrin causes only a moderate reduction in TACC3 levels on spindle MTs [35]. While TACC3^S574A^, TACC3^DEL^ and TACC3^F543A^ all showed significantly reduced levels on mitotic spindles, they were nearly as efficient as the wild-type protein to localize to nascent MTs following MT depolymerisation and regrowth. Importantly, all three mutants contain an intact ch-TOG-binding domain within their TACC domains. Our findings therefore suggest that in early prometaphase, TACC3 is loaded onto nascent MTs where it promotes MT growth, possibly via interaction with ch-TOG at MT plus ends, and generates centrosomal and acentrosomal MT asters that facilitate kinetochore capture [16,22,65,66]. This function of TACC3 is likely to be independent of clathrin. However, as mitosis progresses, an increasing number of kinetochores get captured by MTs, sequestering TACC3 along with clathrin and ch-TOG into inter-MT bridges.

### TACC3: A local activator of Aurora-A on k-fibres?

Aurora-A can be thought of as an incomplete kinase, requiring phosphorylation and co-factors to adopt a fully ordered structure, which is important for its optimal activity [48,49]. Recent work suggests that allosteric activation of the kinase by TPX2^1-43^ may activate Aurora-A in the absence of autophosphorylation, raising the possibility that allosteric activation by different co-factors could be employed to fine-tune Aurora-A activity in a temporally and spatially controlled manner [49]. Here we uncover that human TACC3 also acts as an allosteric activator of Aurora-A *in vitro* in addition to its well-characterized role as a substrate of the kinase. A similar role has been previously described for the *Xenopus* TACC, Maskin [67]. We mapped this activity on TACC3 to the TACC3act domain and generated a point mutant in human TACC3 (TACC3 F525A) that disrupts binding to and activation of Aurora-A *in vitro*. In chicken cells, the equivalent F543A mutation does not influence the overall levels of Aurora-A activity, yet it impairs TACC3 phosphorylation and localization to the mitotic spindle. While there are alternative explanations for these phenotypes, such as a role for the F543 residue in protecting TACC3 from phosphatase activity, we prefer the explanation that TACC3 is a local activator of Aurora-A, as opposed to TPX2, which is responsible for overall Aurora-A activity. Our working hypothesis is that TACC3 can activate Aurora-A locally, thereby increasing its own affinity to clathrin and subsequently to kinetochore MTs. How the TACC3-clathrin-ch-TOG MT-crosslinking complex accumulates specifically on k-fibres is still unclear. The need for local Aurora-A activation is likely to arise if there is insufficient autophosphorylated Aurora-A. Phosphorylated Aurora-A is mostly confined to centrosomes and spindle poles [7,68], and at these locations Aurora-A could certainly phosphorylate TACC3. However, along k-fibres the quantity of phosphorylated Aurora-A might be limiting, perhaps due to the activity of protein phosphatase 6 (PP6) in the cytoplasm [68]. Therefore, a specific need for TACC3 to act as an activator of the kinase might exist on k-fibres. It remains to be established whether this allosteric activation of Aurora-A serves solely to enhance phosphorylation of S558 on TACC3, or the kinase may have other targets in the vicinity, such as clathrin, ch-TOG, or other proteins associated with spindle MTs.

### A speculative model to explain the role of TACC3 in the timing of mitotic spindle assembly

TACC3 has clathrin-independent and clathrin-dependent roles during mitosis, and its phosphorylation by Aurora-A has distinct functions dependent on the context: enhanced centrosome- and spindle targeting to promote MT assembly by the TACC3-ch-TOG complex [26,27,30,69]; and stabilization of the interaction with clathrin, leading to formation of inter-MT bridges on k-fibres [31–34,36]. TACC3^S574A^ cannot be phosphorylated, hence this protein is less active both in promotion of MT assembly and formation of inter-MT bridges. By contrast, TACC3^F543A^ can be phosphorylated by TPX2-activated Aurora-A and supports MT assembly via the ch-TOG polymerase, but it cannot locally activate Aurora-A, which results in reduced S574 phosphorylation and weak complexation with clathrin. Consequently, TACC3^F543A^ is mostly absent from clathrin-containing high molecular weight inter-MT bridges and is more abundant as a low molecular weight complex with ch-TOG.

F543A cells undergo rapid yet faithful mitosis, approximately 20% faster than wild-type cells. This is an unusual phenotype in the literature, and we are not aware of any reported point mutations with a similar effect. We postulate that this phenotype can be explained by the absence of local Aurora-A activation by TACC3^F543A^, and the consequent reduction in complex formation with clathrin. In normal cells the more TACC3 is integrated into inter-MT bridges, the less TACC3 is available to promote MT assembly. Thus, MT-crosslinking and stabilization of k-fibres may occur at the expense of rapid MT formation, and thus the overall balance of these two pools of TACC3 could impact on timing and efficiency of bipolar spindle assembly (Fig 9B). In F543A cells reduced binding of TACC3^F543A^ to clathrin, resulting from impaired local Aurora-A activation on k-fibres, would lead to excess TACC3^F543A^ being available for MT formation, which may accelerate bipolar spindle assembly. While excess TACC3^S574A^ is also available in S574A cells, this mutant cannot be phosphorylated by Aurora-A and has, therefore, a more limited ability to stimulate MT production by ch-TOG. Consequently, mutating S574 to alanine in TACC3 does not shorten mitotic duration. Our data also suggests that phosphorylation of TACC3 by Aurora-A is important for mitotic fidelity, although it remains unclear whether it is the MT growth promoting or crosslinking activities of TACC3 that contribute to faithful chromosome segregation.

Aurora-A and TACC3 are frequently overexpressed in human cancers, and it has been proposed that this might contribute to tumourigenesis by reducing the fidelity of spindle assembly, thereby increasing the rate of genetic errors [5]. We have identified a binding interface between the two proteins that modulates the rate of spindle assembly in a vertebrate cell line. Interestingly, a number of mutations map to this region in cancer cell lines as well as primary tumours [70], suggesting that deregulation of Aurora-A and TACC3 interaction might benefit the mitotic fitness of cancer cells. The Aurora-A-TACC3 binding interface could therefore be explored as a potential drug target through the generation of protein-protein interaction inhibitors.

## Methods

### Cell culture, drug treatments and transfections

DT40 cells were cultured in suspension in RPMI-1640 medium (Invitrogen) which was supplemented with 10% FBS, 1% chicken serum, 110 U/ml penicillin, 10 mg/ml streptomycin and 50 μM β-mercaptoethanol at 40°C with 5% CO_2_. Nocodazole (Sigma-Aldrich) was used at 1 μg/ml for microtubule regrowth experiment. To induce mitotic arrest DT40 cells were treated with 5 nM of paclitaxel and 100 ng/ml of nocodazole for 6 hours. For immunoprecipitation experiments in Fig 6A–6C and Fig 6E cells were treated with 100 ng/ml nocodazole for 16 hours. Cell lysates were prepared from nocodazole-blocked cells (Fig 6A), or from cells released from nocodazole block for 20 minutes (Fig 6B and 6E and S8A Fig), or from cells that were incubated with 100 nM MG132 (Sigma) for 1 hour following release from nocodazole block (Fig 6A and 6C). For Aurora-A kinase inhibition, cells were incubated with 1 μM MLN8054 (Selleckchem) for 2 hrs. For live imaging experiments DT40 cells were transfected with GFP-tubulin or EB3-GFP (a kind gift of A. Akhmanova) using Neon electroporation system (Life Technologies) according to the manufacturer’s instructions.

### Proliferation assay

Genomic DNA was extracted using phenol-chloroform followed by ethanol precipitation. 1 μg of extracted DNA was then incubated with 5 U of DNA Degradase Plus (Zymo) according to manufacturer's protocol. Analysis was performed using HPLC-MS/MS as described in Bachman et al [56].

### Cloning, protein expression and purification

Full-length human TACC3 was PCR amplified from cDNA as stated for TACC3 629–838 in earlier work [36]. Full-length TACC3 was subcloned into pET-44C3 to allow expression with a N-terminal NusA-tag. A C-terminal non-cleavable His-tag was appended to the coding sequence of full-length TACC3 by primer extension PCR. The expression construct for TACC3act-H6c was produced by the same method. Truncates of TACC3 were subcloned from full-length TACC3 cDNA into pETM6T1 for expression with an N-terminal His-NusA-tag. Site-directed mutagenesis was carried out to introduce a stop codon at residue 547 within pETM6T1 TACC3act to produce the expression construct for TACC3 519–546. Point mutations and internal deletion TACC3 constructs were generated by site-directed mutagenesis. The expression construct, pGEX-cs TPX2^1-43^ was produced in previous work [48] and subcloned into pET30TEV for expression with an N-terminal His-tag.

The vectors, pETM11 Aurora-A 122–403, pET30TEV Aurora-A 122–403 D274N (AurA-DN) and pET30TEV Aurora-A 122–403 C290A, C393A were generated in previous work [48,54]. AurA 1–129 and AurA-DN were subcloned into pGEX-6P1 and pGEX-cs, respectively for expression with a N-terminal GST-tag. The vector, pGEX-2T was used for the expression of GST.

Lambda phosphatase was cloned into pCDF-Duet as stated previously [71]. The expression vector for PP1α was a gift from Prof. David Barford (MRC Laboratory of Molecular Biology, Cambridge, UK). All constructs were confirmed by restriction digest and DNA sequencing (GATC; Eurofins MWG Operon).

All human TACC3 constructs in pETM6T1 were expressed and purified as previously described [36]. All proteins were either dialysed or subject to size-exclusion chromatography (SEC) into 50 mM Tris pH 7.5, 150 mM NaCl, 1 mM MgCl_2_ & 5 mM β-mercaptoethanol prior to storage at -80°C. ^15^N- and ^15^N/^13^C-labelled TACC3act was expressed following the Marley protocol [72]. Cultures were induced with 0.6 mM IPTG and incubated overnight at 21°C prior to harvesting. Labelled TACC3act was purified as stated above and subject to SEC on a HiLoad 16/600 Superdex 200 pg column (GE Healthcare) equilibrated in 20 mM potassium phosphate pH 7.0, 50 mM NaCl, 1 mM DTT & 0.02% (w/v) sodium azide (NMR buffer). TPX2^1-43^ was expressed and purified as previously described (42). In the final SEC step, the protein was exchanged into NMR buffer. PP1α was produced as stated previously [73]. Aurora-A 122–403 (alone or co-expressed with lambda phosphatase), 122–403 D274N and 122–403 C290A, C393A were expressed and purified as described earlier [48]. Chelating Sepharose was used in replace of TALON resin using conditions recommended by the manufacturer (GE Healthcare). GST-AurA 1–129, GST-AurA-DN and GST were expressed under the same conditions and purified by affinity chromatography using Gluthathione Sepharose 4B as per the manufacturer’s instructions (GE Healthcare). All Aurora-A proteins were subject to a final SEC step using a HiLoad 16/600 Superdex 200 column (GE Healthcare) equilibrated in 20 mM Tris pH 7.0, 200 mM NaCl, 5 mM MgCl_2_, 5 mM β-mercaptoethanol and 10% glycerol. For NMR studies, AurA-DN was dialysed into NMR buffer overnight. The protein was concentrated to ~500 μM and 5 mM ADP/MgCl_2_ was added to increase kinase stability.

XTACC3 cDNAs were cloned into pGEX4T-1 and *Xenopus* Aurora A cDNAs into pET-28a. Recombinant *Xenopus* proteins were expressed and purified as previously described [15,26]. GST-TPX2^1-39^ recombinant protein was expressed and purified as previously described [48].

### Immunoprecipitation and co-precipitation assays

DT40 cells were lysed in 20 mM sodium phosphate buffer pH 7.4, 150 mM sodium chloride, 2 mM EGTA, 2 mM MgCl_2_, 0.5% Triton X100, 1 mM DTT, 20 mM sodium fluoride, 5 μM Microcystin-LR (Enzo Lifesciences; ALX-350-012) and protease inhibitors (Sigma, P8340) followed by centrifugation at 16,000g for 15 min at 4°C. Lysates were separated on NuPAGE Novex 4–12% Bis-Tris gels (Life Technologies) and transferred onto nitrocellulose membrane for Western blot analysis. For each 200 μl IP reaction, 9 μg of mouse anti-Aurora A (Sigma, A1231), rabbit anti-clathrin heavy chain (Abcam, ab21679) or rabbit anti-chickenTACC3 (60) antibody was cross-linked on 30 μl of Dynabeads Protein G (Life Technologies Ltd, 10003D) and blocked with 5% BSA in PBS with 0.05% Tween20. Mouse (Sigma, I5381) or rabbit (Sigma, I5006) serum IgG was used as control. DT40 wild-type or TACC3 variant cells were treated with 100 ng/ml of nocodazole for 16 hrs and released for 20 min before harvesting them. Cells were lysed on ice for 15 min in 50 mM sodium phosphate buffer pH 7.4, 150 mM sodium chloride, 2 mM EGTA, 1 mM MgCl_2_, 0.5% NP40, 1 mM DTT, 20 mM sodium fluoride, 5% glycerol, 5 μM Microcystin-LR, phosSTOP (Roche, 04906845001) and protease inhibitors followed by centrifugation at 16,000g for 15 min at 4°C. The lysates were pre-cleared with 50 μl of Dynabeads Protein G for 1 hr on a rotary mixer at 4°C. 600 μg of total protein was used per reaction and binding was carried out at 4°C for 1 hr on a rotary mixer. The beads were washed in the above buffer and bound proteins were eluted using 25 μl of 100 mM glycine pH 2.2 followed by neutralization with 5 μl of 1M Tris pH 8. A quarter of the eluted sample was loaded on the gel and processed for Western analysis using mouse anti-clathrin heavy chain (BD Biosciences, 610500), mouse anti-Aurora A, rabbit anti-chicken TACC3 and rabbit phospho-TACC3 (S558) (Cell Signaling, 8842) antibodies. HRP-conjugated goat anti-mouse or anti-rabbit (Dako, P0447 and P0448, respectively) secondary IgGs were used and proteins were detected by chemiluminescence with ECL Western blotting substrate (Thermo, 32106) or SuperSignal West Femto (Thermo, 34094).

100 μg GST-AurA 1–129, GST-AurA-DN or GST was immobilized on 20 μl Gluthathione Sepharose 4B (GE Healthcare) beads equilibrated in 50 mM Tris pH 7.5, 150 mM NaCl, 5 mM MgCl_2_, 5 mM β-mercaptoethanol and 0.02% TWEEN 20. The proteins were incubated with resin for 2 hrs at 4°C. The resin was pelleted by centrifugation and washed twice with buffer. The beads were resuspended in buffer to which 100 μg TACC3 or TPX2^1-43^ was added and incubated for a further 2 hrs at 4°C. The reactions were washed twice with buffer prior to the addition of 20 μl SDS-loading buffer. The reactions were separated by SDS-PAGE. Western blots were performed using an anti-His_6_-tag antibody (1:5000 dilution, Clontech). For co-precipitation assays performed with a gradient of 0–50 μM His_6_-TPX2^1-43^, 2 μM GST-AurA-DN was immobilized on Gluthathione Sepharose 4B beads and 5 μM TACC3act-H6c was used in reactions and performed as above.

### Optimisation of Aurora-A conditions for NMR studies

High concentrations of Aurora-A 122–403 can be easily produced for crystallization trials. However, difficulties were encountered when trying to generate a sample of the kinase in a buffer amenable to NMR spectroscopy at a high protein concentration (~500 μM) for interaction studies with TACC3act. NMR studies were in progress with labelled TACCact in 20 mM potassium phosphate pH 7.0, 50 mM NaCl, 1 mM DTT and 0.02% sodium azide (NMR buffer). The absence of glycerol in the NMR buffer resulted in significant kinase precipitation within hours at room temperature making this unsuitable for NMR spectroscopy as data collection occurs over a number of days. The high level of precipitation upon exchange of Aurora-A 122–403 into NMR buffer meant this had to be carried out by dialysis rather than SEC. A comparison of the stability of Aurora-A 122–403 and Aurora-A 122–403 D274N in NMR buffer showed the latter exhibited less precipitation and was selected for further optimisation. NMR buffer trials investigating the effect of salt concentration (50–150 mM) did not reduce kinase precipitation. The effect of nucleotide on kinase stability was investigated by comparing samples incubated at room temperature in the presence of 5 mM adenosine/MgCl_2_, 5 mM ADP/MgCl_2_ or no additive. These nucleotides were selected as they are frequently used in the crystallization of Aurora-A and would likely improve the stability of the kinase by binding in the ATP-binding pocket of the protein. The addition of nucleotide significantly improved the stability of the kinase. ADP/MgCl_2_ was selected over adenosine/MgCl_2_ as the kinase was stable at a higher concentration for a longer period.

### NMR spectroscopy

All spectra were recorded on Bruker Avance spectrometers at 500 and 700MHz fitted with TCI type cryoprobes. The assignment data for TACC3act were obtained using watergate ^15^N HSQC 2D, ^15^N resolved 3D NOESY-HSQC and 3D TOCSY-HSQC spectra recorded on a sample of 0.8 mM ^15^N-labelled TACC3act in NMR buffer at 298K. These were supplemented with 3D watergate HNCACB, HNCO as well as 2D ^13^C gradient coherence selection sensitivity enhanced HSQC, CAN, CON and (H)NCO experiments recorded on a 0.35 mM sample of ^15^N/^13^C-labelled TACC3act. Interaction of ^15^N-labelled TACC3act with unlabelled AurA-DN was monitored by recording ^15^N watergate HSQC or TROSY 2D experiments at temperatures of 283, 298 and 303K and fields of 500 and 700 MHz in NMR buffer augmented with 5 mM ADP/MgCl_2_. The HSQC/TROSY experiments were supplemented with ^13^CO detected NCO and (H)NCO experiments to combat exchange broadening observed for TACC3act ^1^H resonances in the TACC3act/AurA-DN complex. The concentration range for TACC3act was 100–300 μM and 150–600 μM for AurA-DN. The ternary complex of TACC3act, AurA-DN and TPX2^1-43^ was characterised by 2D watergate ^15^N HSQC, NCO and (H)NCO experiments. To measure binding affinities, titrations were performed with the concentration of TACC3act kept constant at 75 μM and AurA-DN or the complex of AurA-DN/TPX2^1-43^ added at concentrations of 0, 22.5, 45, 75, 150 and 225 μM. All pulse sequences were used as supplied by the spectrometer manufacturer (Bruker) with some in-house modifications. Spectra were processed with Topspin 3.0 and analysed with CCPN analysis 2.1.5 [50]. Sequence specific assignments were obtained using standard procedures in conjunction with ^15^N resolved 3D and triple resonance spectra. Amino acids were identified using a combination of peak patterns in TOCSY spectra and ^13^C chemical shifts. Secondary chemical shifts were calculated within CCPN analysis using the default reference chemical shift library. Binding affinities of TACC3act for AurA-DN and AurA-DN/TPX2^1-43^ were extracted from fitting plots of chemical shift changes for selected amino acids against the concentration of added AurA-DN or AurA-DN/TPX2^1-43^ complex. Fitting was performed using a standard binding isotherm (Eq 1) in R [74]:

fb=b*((v+c+k)-sqrt(abs((v+c+k)^2−4*v*c)))/(2*c) (1)

In Eq (1), the fraction of bound protein is represented by fb, b indicates Bmax, the maximum fraction bound, v is the observed fraction bound and c is the concentration of TACC3act used. k is the dissociation constant, K_d_.

### Microscale thermophoresis

The affinity of Aurora-A to binding partners was quantified by measuring the ratio of unbound Aurora-A:complex at a range of concentrations using MST. This method utillises the differential mobility of a fluorescently-labelled protein and its complexes along a temperature gradient [75]. Aurora-A 122–403 C290A, C393A was labelled with NT-647-NHS dye using a Monolith NT Protein labelling kit as per the manufacturers’ instructions (NanoTemper Technologies GmbH). The kinase was eluted in 50 mM Tris pH 7.6, 150 mM NaCl, 10 mM MgCl_2_, 5 mM β-mercaptoethanol and 0.05% TWEEN 20 (MST buffer) and used at a final concentration of 50 nM. TACC3act constructs were dialysed into MST buffer and used in binding assays at concentrations ranging from 0.2–3950 μM, which were produced by serial dilution of the TACC3act stock. TACC3act and labelled Aurora-A reactions were incubated for 30 mins in the dark at room temperature prior to measurement. Reactions were loaded into Monolith NT Hydrophilic glass capillaries and MST measurements were recorded using a Monolith NT.115 (NanoTemper Technologies GmbH) at 50% LED power and 20% MST power at 25°C. Each capillary was read 3 times. Baselines were subtracted from Temperature Jump data and normalized to fraction bound. Data were averaged for the 3 readings and fit to a one-site specific binding equation (Eq 2) in Prism 6 (GraphPad) to calculate K_d_:

$$\text{y}=\text{Bmax}*\text{x}\;/\;\left({\text{K}}_{\text{d}}+\text{x}\right)$$ (2)

In Eq (2), y is the fraction of TACC3act bound to Aurora-A, Bmax is the maximum fraction bound, x is the concentration of TACC3act and K_d_ is the dissociation constant.

### Homologous gene targeting in DT40 cells

To generate the DEL, F543A and S574A cells sequential gene targeting was employed to edit both alleles of *Tacc3*, which involved homologous recombination of antibiotic resistance genes into intronic regions of the *Tacc3* gene. Cre-lox recombination was then used to excise these cassettes from homozygously targeted cell lines.

To make the targeting construct for generating TACC3 variant cells, left and right homology arms were PCR amplified from DT40 genomic DNA. Please refer to S1 Table for the sequence of oligos.

*TACC3-S574A*: The left arm was amplified by nested PCR and primer extension by TaKaRa LA Taq polymerase (Clontech) using primers SA-LA-fwd, S574A-mutR, S574A-mutF and SA-LA-rev. The amplified PCR product was cloned into pGEM-T vector (Promega), then released as a NotI-SpeI fragment which was then sub-cloned into the same restriction sites in pBluescript II SK(-) containing selection markers (neomycin or blasticidin resistance cassette flanked by LoxP and BamHI sites). The PCR amplified right arm using Phusion DNA polymerase (New England Biolabs) with primers SA-RA-fwd and SA-RA-rev was then inserted as a blunt-end fragment into the above construct that was digested with SmaI. The neomycin resistance cassette in the final construct was then swapped with a puromycin resistance cassette by BamHI digestion.

*TACC3-F543A*: The left arm was PCR amplified by Phusion DNA polymerase using primers FA-LA-SalI-fwd and FA-LA-BamHI-rev followed by cloning into pJET1.2 as blunt-end fragment. Mutagenesis (F543A) was carried out on this construct by inverse PCR using primers F543A-mutF and F543A-mutR that also incorporate a NheI restriction site through silent mutation. The right arm was PCR amplified using primers FA-RA-BamHI-fwd and FA-RA-NotI-rev followed by cloning into pJET1.2. The final construct was prepared by sequential sub-cloning of SalI-left arm-F543A-BamHI followed by BamHI-right arm-NotI and finally neomycin or puromycin resistance cassette as a BamHI fragment.

*TACC3-DEL*: Primers used to amplify the left arm were DEL-LA-KpnI-fwd and DEL-LA-BamHI-STOP-rev and the right arm were DEL-RA-BamHI-fwd and DEL-RA-NotI-rev. Homology arms were cloned into pBluescript II SK(-), with the left arm between KpnI and BamHI sites and the right arm between BamHI and NotI sites. Selection markers (puromycin or blasticidin resistance cassette) were cloned into these vectors between BamHI sites. Restriction digestions and DNA sequencing confirmed all the constructs. Two rounds of targeting to modify the two alleles were performed by electroporation using a gene pulsar (Bio-Rad Laboratories). In brief, 60 μg of linearized DNA was mixed with 2 × 10^7^ cells in chilled PBS in a 4-mm cuvette and electroporated at 550 V and 25 μF, followed by dilution into six 96-well plates. After 24 h, antibiotics were added for 7–10 days at the following concentrations: 1.5 mg/ml neomycin (Invitrogen), 0.5 μg/ml puromycin (Acros Organics), and 50 μg/ml blasticidin (Acros Organics). Antibiotic-resistant clones were expanded, and genomic DNA was extracted. Targeted integrations were screened by PCR amplification using the primers given in S1 Table (see S4–S6 Figs). Positive clones were subsequently electroporated with Cre-recombinase encoding plasmid to excise out the antibiotic resistance cassettes from the gene alleles. cDNA sequencing validated the final clones.

### *Xenopus* egg extract and pulldowns

Cytosolic-factor-arrested *Xenopus* egg extracts (CSF extract) and GST-pull downs were performed as previously described [26]. In short, 20 μl protein A-conjugated Dynabeads 280 (Invitrogen) were washed 3 times with PBS-Triton (0.1%), incubated for 30 mins at room temperature with anti-GST antibodies and washed twice with PBS-Triton (0.1%) and twice with CSF-XB (10 mM Hepes, 100 mM KCl, 0.1 mM CaCl_2_, 3 mM MgCl_2_, 50 mM Sucrose, 5mM EGTA pH to 7.7 with KOH). Beads were then incubated for 60 mins on ice with 60 μl of CSF-egg extract preincubated with 3 μM of recombinant protein for 15 mins at 20°C. Beads were retrieved and washed twice with CSF-XB and twice with PBS-Triton (0.1%), in the presence of phosphatase inhibitors. Proteins were eluted with Laemmli buffer and analyzed by Western blot.

For *in vitro* GST-pull downs, anti-GST coated beads prepared as above were incubated with 5–10 μg of GST recombinant proteins. Beads were retrieved, washed and incubated with 0.25 μM His-AurA in kinase buffer (20 mM Hepes pH 7.5, 200 mM KCl, 30 mM MgCl_2_, 0.5 mM EGTA, 1 mM DTT, 0.05% Triton X-100, 0.2% β-mercaptoethanol, and 1 mg/ml BSA) for 30 mins at 22°C. Beads were retrieved and washed three times with PBS, 0.5% Triton X-100, 0.25 mM Na_3_VO_3_, 10 mM NaF. Proteins were eluted with Laemmli buffer and analyzed by Western blot.

### Sequence alignments

Multiple sequence alignments of *Homo sapiens* (NCBI accession code: NP_006333), *Gallus gallus* (NCBI accession code: NP_001004429.2), *Xenopus laevis* (NCBI accession code: NP_001081964.1) and *Mus musculus* (NCBI accession code: NP_001035525.1) TACC3 were performed using ClustalW2 [76].

### *In vitro* kinase assays

Kinase assays performed with recombinant TACC3 consisted of 0.625 μM Aurora-A 122–403, 5 μM TACC3 and/or 0.25 mg/ml MBP (Sigma). Reactions were carried out in kinase buffer (20 mM Tris pH 7.5, 25 mM NaCl, 1 mM MgCl_2_, 1 mM DTT & 0.01% TWEEN 20). Reactions were initiated by the addition of ^32^P-ATP (Perkin Elmer), incubated at room temperature for 10 mins and were analysed by either SDS-PAGE followed by autoradiography or scintillation counting, in which case reactions were stopped by the addition of 2% orthophosphoric acid (Sigma). Samples were transferred onto P81 paper (Whatman), unincorporated ^32^P-ATP removed by extensive washing with 0.2% orthophosphoric acid and incorporation of ^32^P quantified. Statistical analysis was carried in Prism (Graphpad Software, Inc.). One-way ANOVA was performed to identify significant changes followed by Dunnett’s post-hoc analysis. Kinase reactions for analysis by quantitative immunofluorescent Western blot were performed with cold ATP only. Analysis was carried out as per the manufacturer’s instructions (LI-COR GmbH) using an anti-phospho-S558-TACC3 antibody (1:1000 dilution [77]) and IRDye 800CW secondary antibody (LI-COR GmbH). Blots were resolved using Image Studio on an Odyssey CLx Infrared imaging system (LI-COR GmbH). Statistical analysis was carried in Prism (Graphpad Software, Inc.). One-way ANOVA was performed to identify significant changes followed by Dunnett’s post-hoc analysis.

To analyse endogenous Aurora-A activity in WT, F543A and DEL cells, Aurora-A was immunoprecipitated from the corresponding cell extracts. Beads were retrieved on a magnet, washed twice with 500 μl of lysis buffer (20 mM sodium phosphate buffer pH 7.4, 150 mM sodium chloride, 2 mM EGTA, 2 mM MgCl_2_, 0.5% Triton X100, 1 mM DTT, 20 mM sodium fluoride, 5 μM Microcystin-LR (Enzo Lifesciences; ALX-350-012) and protease inhibitors (Sigma, P8340)) and twice with kinase buffer and the assay was performed as described above.

### Aurora-A autophosphorylation assays

*In vitro* kinase assays were performed (as above with cold ATP) with Aurora-A 122–403 co-expressed with lambda phosphatase alone and on addition of TACC3act and TPX2^1-43^. Reactions were quenched by the addition of SDS-loading buffer and separated by SDS-PAGE. Western blots were performed using an anti-Phospho-Thr288 Aurora-A antibody (1:1000 dilution, Cell Signaling Technology).

### Aurora-A/PP1α dephosphorylation assays

50 μM Aurora-A 122–403 was incubated with 0.1 μM PP1α alone and in the presence of 10 μM TACC3act or TPX2^1-43^ for 1 hr at room temperature. Assays were carried out in 50 mM Tris pH 7.5, 0.1 mM EDTA, 2 mM MnCl_2_, 5 mM DTT and 0.025% TWEEN 20. Reactions were stopped by the addition of SDS-loading buffer and separated by SDS-PAGE. Western blots were performed using an anti-Phospho-Thr288 Aurora-A antibody (1:1000 dilution, Cell Signaling Technology).

### Density gradient ultracentrifugation

DT40 cells were treated with 100 ng/ ml of nocodazole for 16 hrs and released for 20 min before harvesting. Cells were lysed on ice for 15 min in 50 mM sodium phosphate buffer pH 7.4, 150 mM sodium chloride, 2 mM EGTA, 1 mM MgCl2, 0.5% NP40, 1 mM DTT, 20 mM sodium fluoride, phosSTOP (Roche) and protease inhibitors (Sigma) followed by centrifugation at 16,000g at 4°C for 15 min. A 5 to 40% (w/v) step sucrose gradient (900 μl each) was prepared in the above buffer and layered on 900 μl of a 2M sucrose cushion (approx. 70%) in Beckman Coulter Ultra-Clear 14 ml tubes. 5 mg of total cell lysate in 900 μl was loaded on the gradient and subjected to ultracentrifugation using SW40Ti rotor at 23,700 rpm for 16 hr at 4°C in a Beckman Coulter Optima L-100 XP ultracentrifuge. 450 μl fractions were collected and TCA-precipitated before being subjected to western blot analyses.

### Antibodies and immunostainings

Primary antibodies used in this study were anti-CDK5RAP2 [78], anti-TACC3 against aa 126–442 of *Gallus gallus* TACC3 [78], anti-phospho-S558-TACC3/P-TACC3 (Cell Signaling), anti-ch-TOG (QED Bioscience, 34032), anti-Clathrin Heavy Chain (Abcam; 21679 and BD Biosciences, 610500), anti-Aurora-A (35C1; Sigma), anti-α-tubulin (Dm1α; Sigma), anti-α-tubulin-FITC (Sigma), anti-γ-tubulin (GTU88; Sigma) anti-phospho-Histone H3 (Millipore), anti-BubR1 (kind gift of W. Earnshaw) and phospho-Aurora-A/B/C (Cell Signaling). For visualization of mitotic spindles and centrosomal proteins, cells were fixed and immunostained as described in [79]. P-TACC3 antibody staining was carried out in cells fixed in 4% paraformaldehyde (PFA) in PBS. For clathrin antibody, cells were fixed in warm 3% PFA in PHEM buffer (60 mM Pipes, 25 mM Hepes, 10 mM EGTA, 2 mM MgCl_2_ pH 6.8) for 15 mins, followed by extraction with PBS/0.5% TritonX-100 for 15 mins.

### Image acquisition, processing and quantification

Imaging of fixed cells was performed on a scanning confocal microscope (Eclipse 90i; Nikon, Leica SP5 or Nikon A1). Cells were mounted in anti-fade medium (ProLong Gold or Mowiol 4–88) and imaged with 100X, 1.4 NA objective (Nikon) or 60X, 1.4 NA objective (Nikon or Leica). Images presented here are 3D projections of z sections taken every 0.5 μm across the cell. Images of any individual figures were acquired using the same settings and were imported into Volocity 6.3 (Perkin Elmer) or Photoshop CS6 (Adobe). Time-lapse images of DT40 cells expressing GFP–α-tubulin or EB3-GFP were acquired as described in [79]. Images were acquired every 3 min for 2–3 hrs. For quantifications of TACC3 spindle intensity and spindle length, non-saturated images of randomly selected mitotic cells were taken using identical acquisition settings. Images were uploaded in Volocity and 3D measurements were performed using analysis protocols allowing batch processing. For TACC3 and clathrin intensity quantification, fluorescence intensities were measured within two half spindle volumes as determined by tubulin fluorescence. The 3D object definition was improved using de-noising and size filters. TACC3 and clathrin intensity values were normalized against tubulin intensity.

For spindle length quantification, the centrosome distance was measured in mitotic cells using a fully automated protocol. Three populations of 3D objects were defined as “nuclei”, “mitotic” and “centrosomes” and detected using DNA, phospho-histone H3 and γ-tubulin staining, respectively. The proper segmentation of the nuclei was achieved using the “separate touching object” tool. Fine filter de-noising was used to improve definition of phospho-histone H3 objects. We then used the compartmentalizing tool to associate “mitotic” and “centrosomes” objects to individual “nuclei” objects in order to only measure the 3D distance between centrosomes belonging to the same cell. As false positive or false negative objects could be detected within the “centrosomes” population, we filtered the results to only consider nuclei associated with exactly two centrosomes.

### Microtubule pelleting and regrowth

For the microtubule pelleting experiment, cells were lysed in 50 mM Tris pH 7.4, 5 mM MgCl_2,_ 0.1 mM EGTA and 0.5% Triton X-100 (to a final total protein of 2.5 mg). Cell lysate was incubated for 5 min at 30°C followed by pre-clearing by centrifugation at 70,000 rpm for 10 minutes at 40°C. Paclitaxel-stabilized microtubules were prepared in BRB80 buffer (80 mM PIPES-K pH 6.8, 1 mM EGTA, 1 mM MgCl_2_) and added to the cell lysate (250 ng). In control samples the same amount of tubulin was added without paclitaxel. After 30 minutes at 30°C, the samples were centrifuged at 60,000 rpm for 20 min at 30°C in a TLA-100 rotor through a 1 M sucrose cushion in BRB80 buffer. The pellets were washed three times with warm BRB80 and analyzed by Western blotting. For microtubule regrowth experiments, cells were treated as described in [79]. Cells on coverslip were fixed for immunostaining after incubation for 3 and 15 min at 40°C to polymerise microtubules.

### Metaphase chromosome spreads

DT40 cells were processed as described in [79].

### shRNA in Jurkat cells

Jurkat E6.1 cell line was grown in RPMI1640 containing 5% FBS supplemented with penicillin and streptomycin. TACC3 shRNA constructs were designed, prepared and transduced into Jurkat cells as described in [80]. shRNAs sequences are shown in S1 Table. The shRNA encoding fragments were cloned into XhoI-EcoRI sites in MSCV-miR30-puro vector.

### Statistical analysis

Statistical analysis and graphs were carried out using Prism (Graphpad Software, Inc.). Numbers of experimental repeats (n values) are reported for each dataset in figures and figure legends. T-test was performed on all data with normal distribution. When normal distribution could not be confirmed, the non-parametric Mann-Whitney test was used.

## Supporting Information

S1 Fig SDS-PAGE analysis of TACC3 protein fragments and SEC and NMR analysis of TACC3act on interaction with AurA-DN and TPX2^1-43^. (A) TACC3 proteins used for *in vitro* kinase activity assays were resolved by SDS-PAGE. Asterisks mark the full-length protein for each TACC3 construct. (B) SEC performed on AurA-DN C290A, C393A, TPX2^1-43^ and TACC3act alone and in complex. Chromatographs observed on gel filtration of the proteins on a HiLoad Superdex 200 X16/60 column are shown above. Fractions across the elution volume were subject to SDS-PAGE analysis and are shown below. (C) Graphic representation of the chemical shift perturbations (Δδ) observed on interaction between ^15^N-labelled TACC3act and AurA-DN. (D) Graphic representation of the chemical shift perturbations (Δδ) observed on interaction between ^15^N-labelled TACC3act, AurA-DN and TPX2^1-43^.
(PDF)

S2 Fig ^15^N-NMR spectroscopy analysis of TACC3act. (A) 3D HNCACB and ^15^N NOESY-HSQC spectra of TACC3act. The spectra demonstrate the helix specific amide-amide sequential nuclear Overhauser effects (NOEs) for the helical regions of TACC3act. (B) Summary of the secondary specific short range NOE distances and secondary chemical shifts of TACC3act.
(PDF)

S3 Fig Biochemical characterization of *Xenopus* AurA/TACC3 interaction. (A) Co-precipitation assay to assess binding between GST-TACC3 or GST-TACC3–F589A and endogenous AurA in *Xenopus* egg extract using Gluthathione Sepharose beads. GST was used as control. (B) *In vitro* co-precipitation assay to assess binding between GST-XTACC3 and his-AurA. The assay used GST and wild-type, phospho-null (SA) and phospho-mimic (SE) GST-XTACC3 as prey proteins. His-AurA-WT (wild-type), top panel, or His-AurA–KD (kinase dead), bottom panel, were used as prey proteins. (C) Activation of his-AurA by GST-XTACC3 WT, SA and SE was monitored by *in vitro* kinase activity assay. GST tagged-*Xenopus* TPX2^1-39^ was used as a positive control for AurA activation and GST as a negative control. The protein levels are shown in the Coomassie blue stained gels (top). The corresponding autoradiographs are shown below. The chart on the right shows the quantification of the autoradiography signal for HH3 as fold change in respect to the GST alone lane in this representative experiment.
(PDF)

S4 Fig Gene knock-out strategy for generating DEL DT40 cells. (A) Schematic representation of the gene targeting strategies. Exons 6–8 were replaced by antibiotic resistance cassettes flanked by LoxP sites (triangles). (B) Confirmation of gene targeting events by PCR using genomic DNA extracted from WT, DEL-heterozygous and DEL-homozygous cell lines. Block arrows show the position of primers. The antibiotic resistance cassettes were removed by Cre recombinase mediated excision. The targeting affected the splice junctions between exons 5–6 and 8–9 that ultimately resulted in a TACC3 deletion mutant lacking exons 5 to 9, which was confirmed by sequencing the cDNA prepared from the homozygous DEL DT40 cells. This also resulted in the absence of the stop codon in the cDNA, which was introduced at the end of exon 5 in the targeting construct.
(PDF)

S5 Fig Gene knock-in strategy for generating S574A DT40 cells. (A) S574A mutation was incorporated into exon 7 of the left arm of the targeting construct with the antibiotic resistance cassettes flanked by LoxP sites (triangles) introduced into intron 8. (B) Confirmation of gene targeting events by PCR using genomic DNA extracted from WT, S574A- heterozygous and S574A- homozygous cell lines. Block arrows show the position of primers. The antibiotic resistance cassettes were removed by Cre recombinase mediated excision. (C) Sequencing of cDNA prepared from the homozygous TACC3-S574A DT40 cells confirmed the incorporation of the mutation in to the genomic locus.
(PDF)

S6 Fig Gene knock-in strategy for generating F543A DT40 cells. (A) and (B) F543A mutation was incorporated into exon 5 of the left arm of the targeting construct with the antibiotic resistance cassettes flanked by LoxP sites (triangles) introduced into intron 5. (C) Confirmation of gene targeting events by PCR using genomic DNA extracted from WT, F543A- heterozygous and F543A- homozygous cell lines. Block arrows show the position of primers. The antibiotic resistance cassettes were removed by Cre recombinase mediated excision. (D) Sequencing of cDNA prepared from the homozygous TACC3-F543A DT40 cells confirmed the incorporation of the mutation into the genomic locus.
(PDF)

S7 Fig Localisation of TACC3 and chTOG in TACC3 mutant DT40 cells. (A) Anti-TACC3 antibody staining is shown in DT40 cells of various genotypes in G2 (top panels), prometaphase (middle panels) and metaphase (bottom panels). In merged images TACC3 is in red, α-tubulin is green and DNA is blue. (B) TACC3 localisation with respect to the centrosome is shown in DT40 cells of various genotypes. Centrosomes are marked by anti-γ-tubulin antibodies in red, TACC3 is green and DNA is blue. Framed areas are shown at higher magnification below. Note that TACC3-DEL localises weakly to MT minus ends, but not to centrosomes. (C) Levels of the MT polymerase, ch-TOG, are reduced on the mitotic spindle in the TACC3 mutant lines. The extent of ch-TOG reduction correlates with the degree of TACC3 loss from the spindle (Fig 5E) with F543A being the mildest. In merged images α-tubulin is green, ch-TOG is red and DNA is blue. (D) ch-TOG remains associated with the centrosome in the TACC3 mutant cell lines. In merged images γ-tubulin is red, ch-TOG is green and DNA is blue. Box plot depicts overall intensity of ch-TOG staining at centrosomes. Ch-TOG signal intensity was quantified in centrosome volumes defined by γ-tubulin staining. A minimum of 50 centrosomes was scored per genotype. Whiskers in box plot correspond to minimum and maximum values and the boxes to the interquartile range for each genotype. Statistical significance was assessed using Mann Whitney nonparametric t-test (* P < 0.05). Scale bar = 5 μm. (E) MT-pelleting experiments were performed using mutant cell extracts as indicated. MT polymers were obtained by incubating purified tubulin with paclitaxel.
(PDF)

S8 Fig The F525/F543 residue is important for Aurora-A binding, but not for global Aurora-A activity. (A) Western blots show immunoprecipitation of TACC3 from DT40 cell extracts. Genotypes are as indicated. Antibodies against TACC3 or random rabbit IgG (Con) were used for immunoprecipitation (IP). Blots were probed with anti-TACC3 or anti-phospho-S574-TACC3 (P-TACC3) antibodies. Note the absence of P-TACC3 signal in S574A and DEL cells confirming the specificity of the P-TACC3 antibody. We were unable to detect P-TACC3 in the inputs. (B) TACC3^F543A^ is phosphorylated by Aurora-A. Antibodies against phospho-S574 TACC3 (P-TACC3) weakly stain spindle poles of WT cells. Some spindle pole staining is also visible in F543A cells. Note the absence of staining in S574A cells confirming the specificity of the P-TACC3 antibody. In merged images α-tubulin is red, phospho-TACC3 is green and DNA is blue. (C) TACC3 is dispensable for T loop phosphorylation of Aurora-A. A phospho-specific antibody detects phosphorylation at T288 of Aurora A. Mitotic cells expressing varying levels of TACC3 contain similar amounts of phosphorylated Aurora-A. Note that the centrosomal signal corresponds to Aurora-A, whereas signal overlapping the chromatin is likely to reflect phosphorylated Aurora-B, as the antibody also reacts with T-loop phosphorylated Aurora-B. Lower panels show Jurkat cells after treatment with DMSO or the Aurora-A kinase inhibitor MLN8054 (4 M). The phospho-antibody is specific, since staining disappears upon treatment of Jurkat cells with MLN8054. In merged images TACC3 is red, P-Aurora-A is green and DNA is blue. (D) Aurora-A kinase immunoprecipitated from WT and TACC3 mutant cell lines shows similar activity *in vitro*. Aurora-A was immunoprecipitated using anti-Aurora A antibody (Aurora A IP) and random IgG was used as control (control IP). The autoradiography (^32^P-MBP, top panel) corresponds to the Coomassie-stained gel (bottom panel). (E) Aurora-A localizes normally in all three TACC3 mutant cell lines. In merged images the centrosomal marker CDK5RAP2 is green, Aurora-A is red and DNA is blue. (F) Aurora-A interaction with TACC3 is perturbed in the mutants. Western blots show co-immunoprecipitation of Aurora-A and TACC3 from DT40 cell extracts. Genotypes are as indicated. Antibodies against Aurora-A (AurA) or random mouse IgG (Con) were used for immunoprecipitation (IP). Blots were probed with anti-Aurora-A or anti-TACC3 antibodies, as indicated. Graph on right depicts quantification of the TACC3 signal from western blots after normalizing the signal against total immunoprecipitated Aurora-A. a.u. = arbitrary units. Scale bars = 5 μm.
(PDF)

S9 Fig The spindle assembly checkpoint (SAC) is intact in F543A cells. (A) Localisation of the SAC component, BubR1, to kinetochores is comparable between F543A and WT cells. In merged images k-fibres are stained with anti-α-tubulin (red), anti-BubR1 (green) and DNA (blue). (B) F543A cells arrest in mitosis as efficiently as WT. Cells treated for 6 hours with low doses of nocodazole (100 ng/ml) or paclitaxel (5 nM) were analysed. Mitotic index was quantified by Hoechst staining with >500 cells analysed. (C) Lamin B antibody reveals intact nuclear envelope in F543A cells. Lamin B is green, the centrosome marker γ-tubulin is red and DNA is blue. (D) Inter-centrosome distances in the last frame before NEBD are plotted against duration of NEBD-anaphase onset based on time-lapse experiments shown in Fig 7B and 7C. Scale bars = 5 μm.
(PDF)

S1 Table Oligonucleotides used in the generation of DT40 constructs and for shRNA studies. (PDF)

S1 Video Mitosis in a WT DT40 cell transfected with GFP-tubulin. Images were acquired by time-lapse confocal microscopy using a spinning-disc confocal system (PerkinElmer). Frames were acquired every 3 min.
(MOV)

S2 Video Mitosis in a TACC3^F543A^ DT40 cell transfected with GFP-tubulin. Images were acquired by time-lapse confocal microscopy using a spinning-disc confocal system (PerkinElmer). Frames were recorded every 3 min.
(MOV)

S3 Video Mitosis in a TACC3^DEL^ DT40 cell transfected with GFP-tubulin showing a multipolar spindle. Images were acquired by time-lapse confocal microscopy using a spinning-disc confocal system (PerkinElmer). Frames were recorded every 3 min.
(MOV)

S4 Video Mitosis in a TACC3^DEL^ DT40 cell transfected with GFP-tubulin showing a “poorly organized spindle”. Images were acquired by time-lapse confocal microscopy using a spinning-disc confocal system (PerkinElmer). Frames were recorded every 3 min.
(MOV)
