# Supplementary figures and images for: Aurora-A-Dependent Control of TACC3 Influences the Rate of Mitotic Spindle Assembly
Source: PLoS Genet. 2015 Jul 2;11(7):e1005345. doi: 10.1371/journal.pgen.1005345 (PMC4489650; doi:10.1371/journal.pgen.1005345)

**Figure S1.**

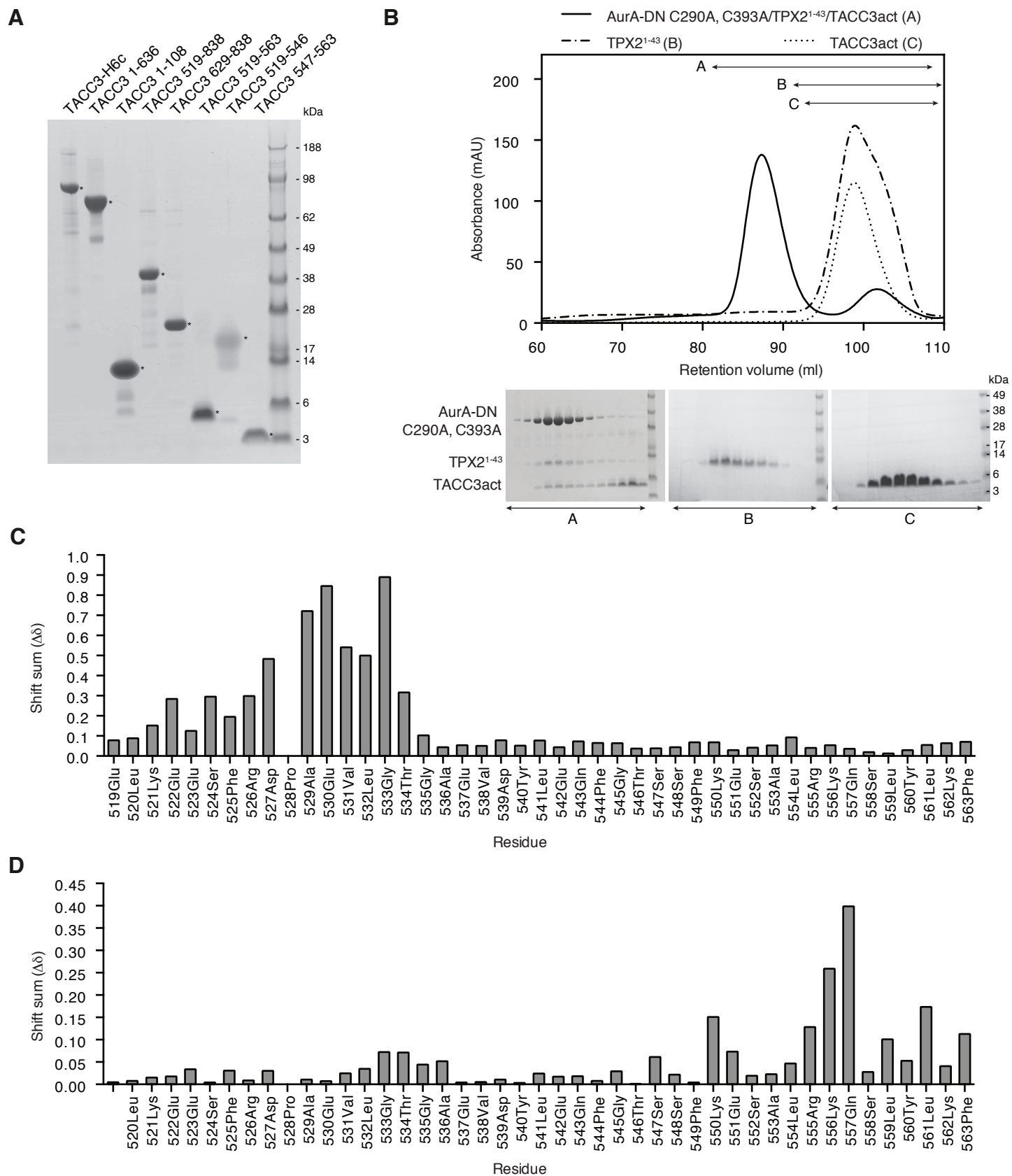

Supplement: S1 Fig — (A) TACC3 proteins used for in vitro kinase activity assays were resolved by SDS-PAGE. Asterisks mark the full-length protein for each TACC3 construct. (B) SEC performed on AurA-DN C290A, C393A, TPX21-43 and TACC3act alone and in complex. Chromatographs observed on gel filtration of the proteins on a HiLoad Superdex 200 X16/60 column are shown above. Fractions across the elution volume were subject to SDS-PAGE analysis and are shown below. (C) Graphic representation of the chemical shift perturbations (Δδ) observed on interaction between 15N-labelled TACC3act and AurA-DN. (D) Graphic representation of the chemical shift perturbations (Δδ) observed on interaction between 15N-labelled TACC3act, AurA-DN and TPX21-43. (PDF) [file pgen.1005345.s001.pdf]

Figure S2.

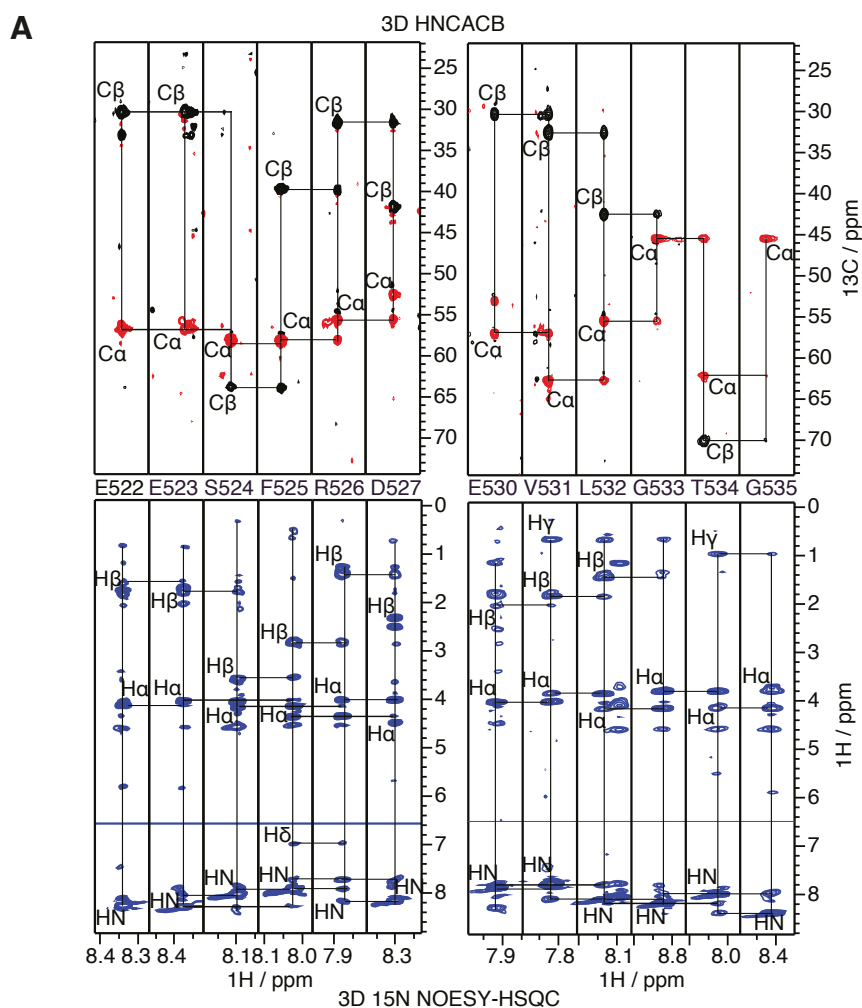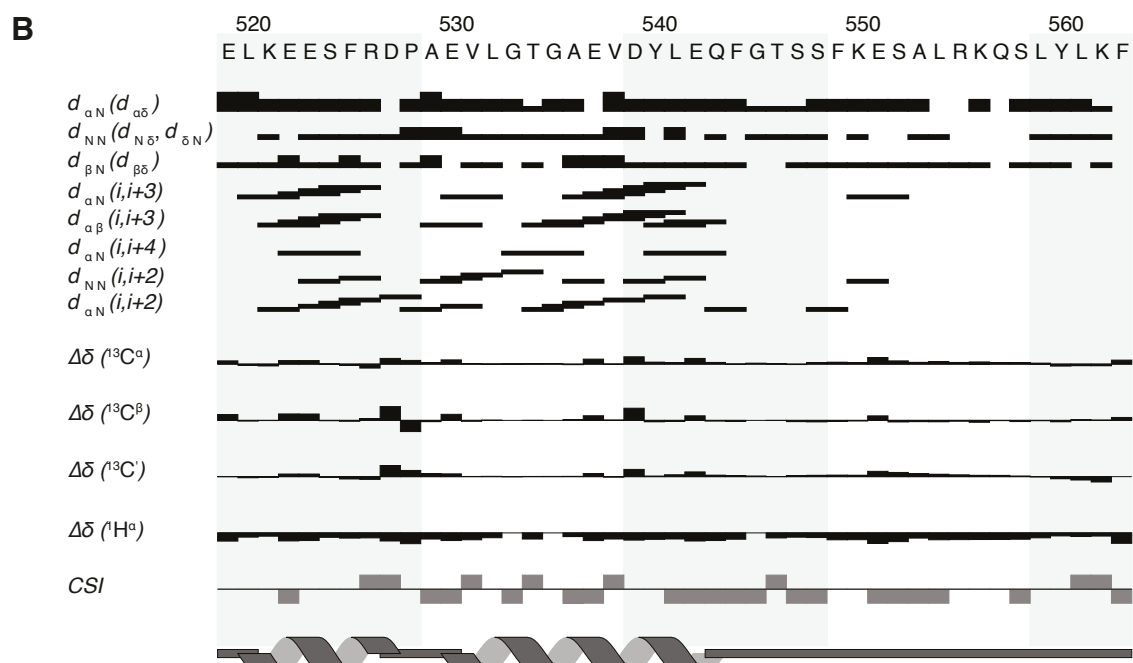

Supplement: S2 Fig — (A) 3D HNCACB and 15N NOESY-HSQC spectra of TACC3act. The spectra demonstrate the helix specific amide-amide sequential nuclear Overhauser effects (NOEs) for the helical regions of TACC3act. (B) Summary of the secondary specific short range NOE distances and secondary chemical shifts of TACC3act. (PDF) [file pgen.1005345.s002.pdf]

**Figure S3**

**A**

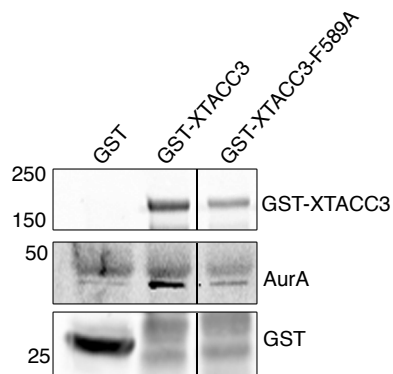

**B**

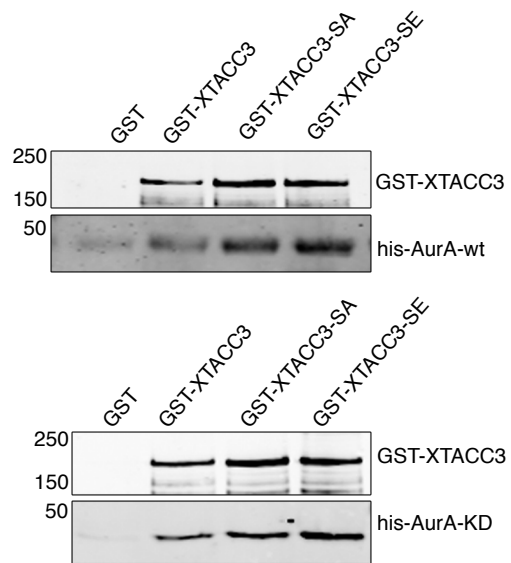

**C**

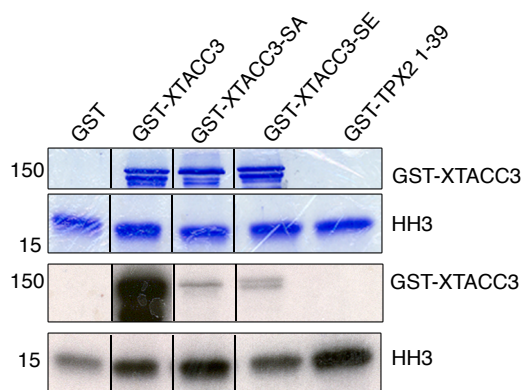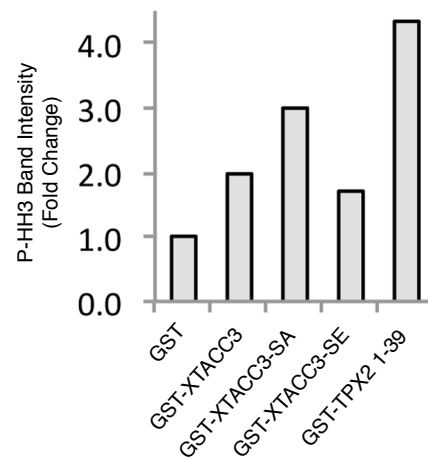

Supplement: S3 Fig — (A) Co-precipitation assay to assess binding between GST-TACC3 or GST-TACC3–F589A and endogenous AurA in Xenopus egg extract using Gluthathione Sepharose beads. GST was used as control. (B) In vitro co-precipitation assay to assess binding between GST-XTACC3 and his-AurA. The assay used GST and wild-type, phospho-null (SA) and phospho-mimic (SE) GST-XTACC3 as prey proteins. His-AurA-WT (wild-type), top panel, or His-AurA–KD (kinase dead), bottom panel, were used as prey proteins. (C) Activation of his-AurA by GST-XTACC3 WT, SA and SE was monitored by in vitro kinase activity assay. GST tagged-Xenopus TPX21-39 was used as a positive control for AurA activation and GST as a negative control. The protein levels are shown in the Coomassie blue stained gels (top). The corresponding autoradiographs are shown below. The chart on the right shows the quantification of the autoradiography signal for HH3 as fold change in respect to the GST alone lane in this representative experiment. (PDF) [file pgen.1005345.s003.pdf]

**Figure S4.**

**A**

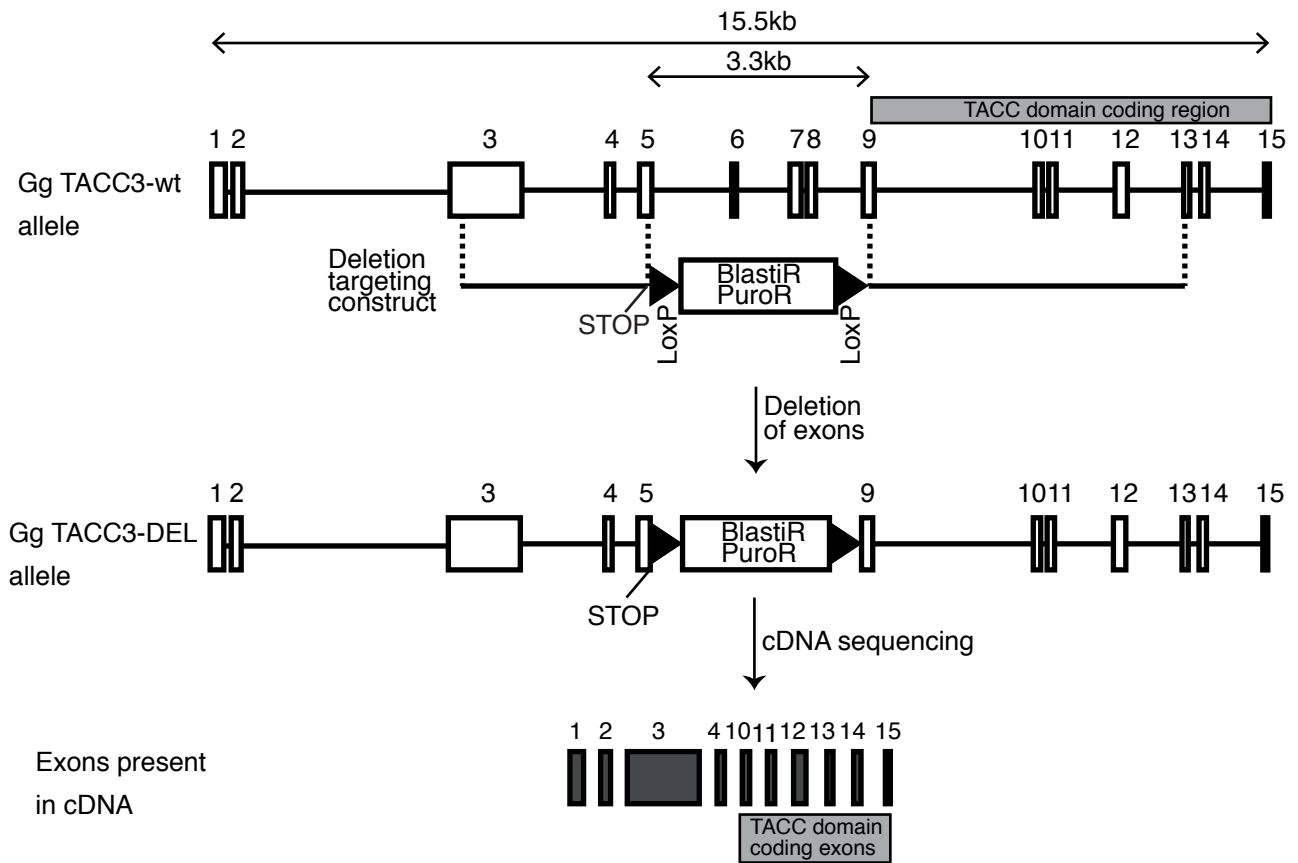

**B**

**Targeted genomic locus**

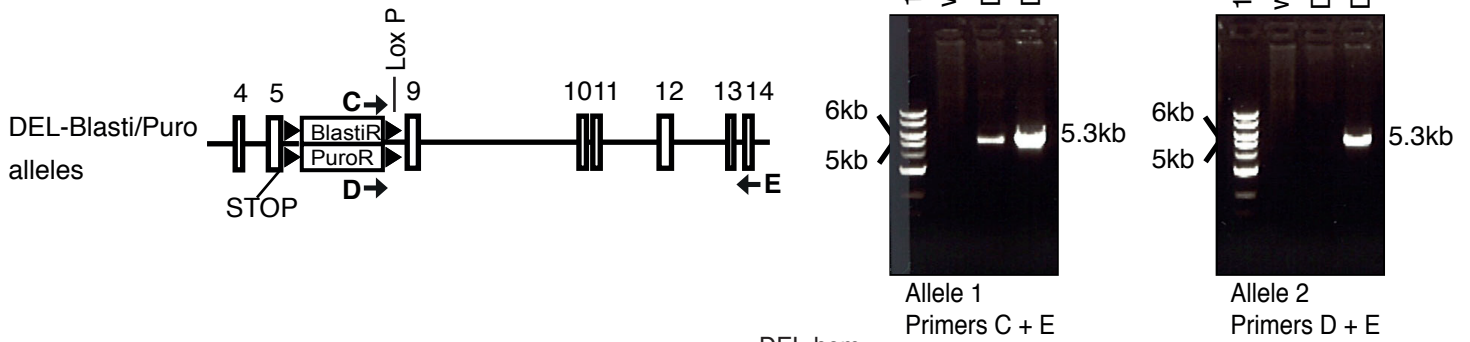

**Targeted genomic locus after Cre-excision**

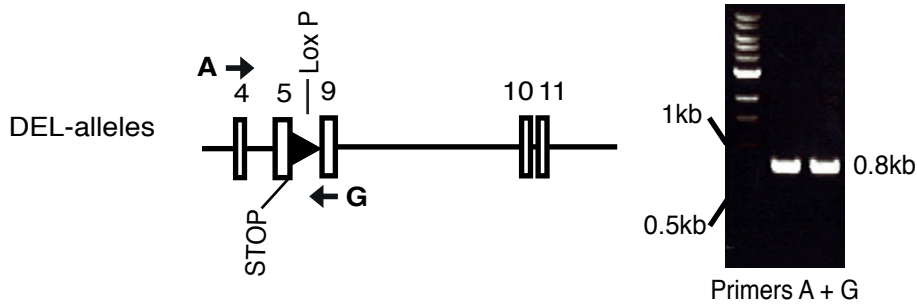

Supplement: S4 Fig — (A) Schematic representation of the gene targeting strategies. Exons 6–8 were replaced by antibiotic resistance cassettes flanked by LoxP sites (triangles). (B) Confirmation of gene targeting events by PCR using genomic DNA extracted from WT, DEL-heterozygous and DEL-homozygous cell lines. Block arrows show the position of primers. The antibiotic resistance cassettes were removed by Cre recombinase mediated excision. The targeting affected the splice junctions between exons 5–6 and 8–9 that ultimately resulted in a TACC3 deletion mutant lacking exons 5 to 9, which was confirmed by sequencing the cDNA prepared from the homozygous DEL DT40 cells. This also resulted in the absence of the stop codon in the cDNA, which was introduced at the end of exon 5 in the targeting construct. (PDF) [file pgen.1005345.s004.pdf]

**Figure S5.**

**A**

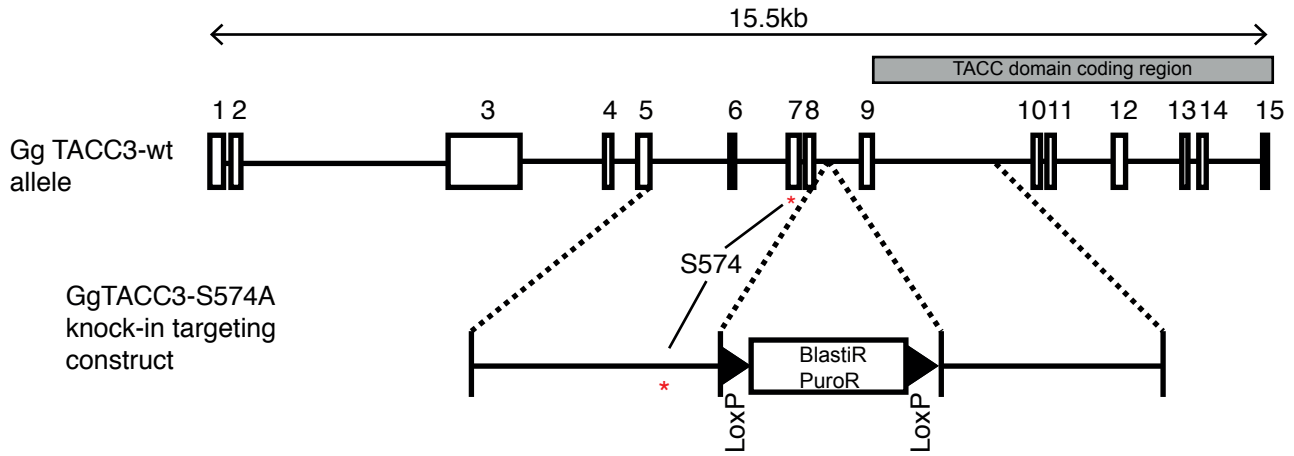

**B**

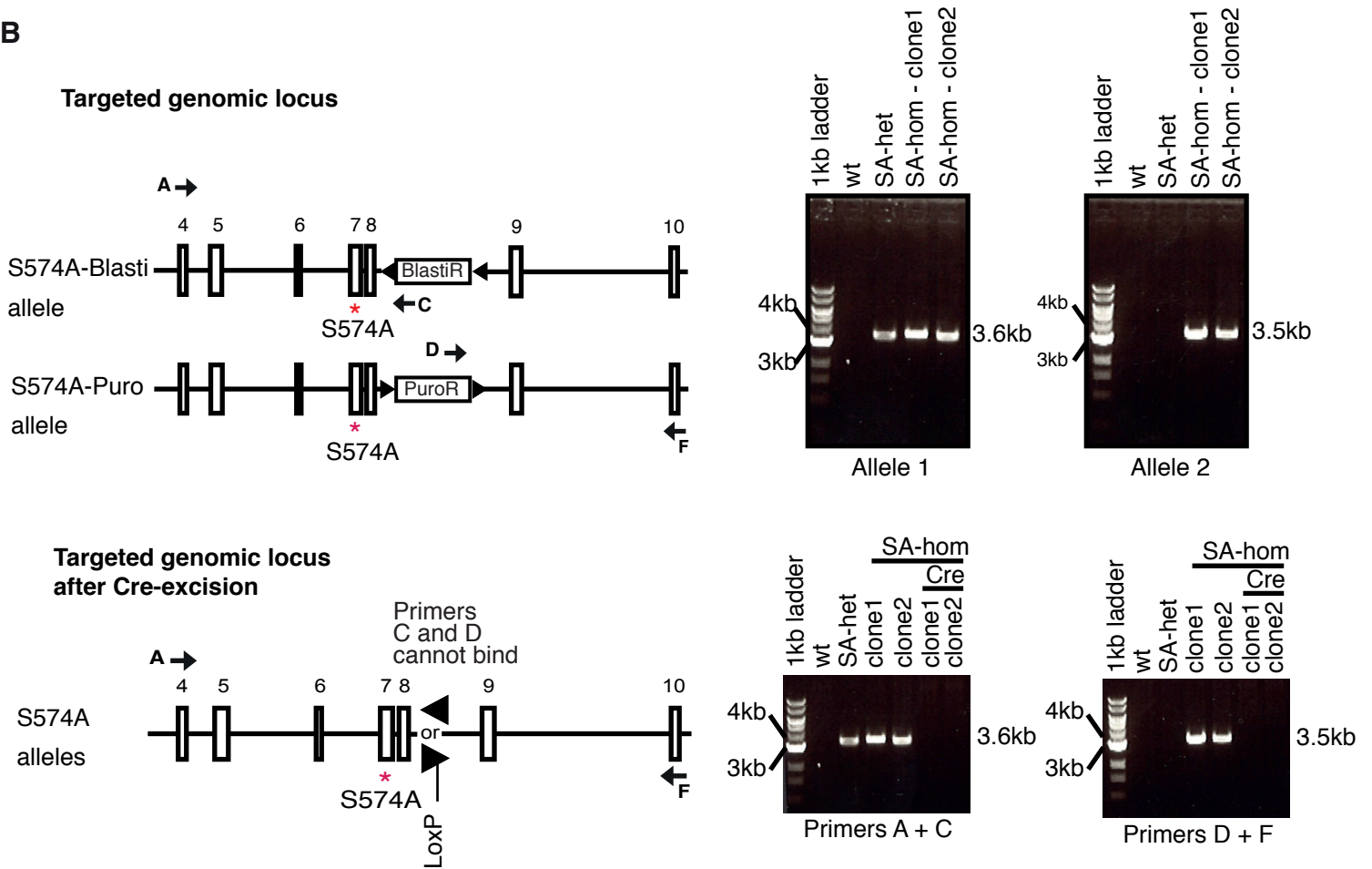

**C**

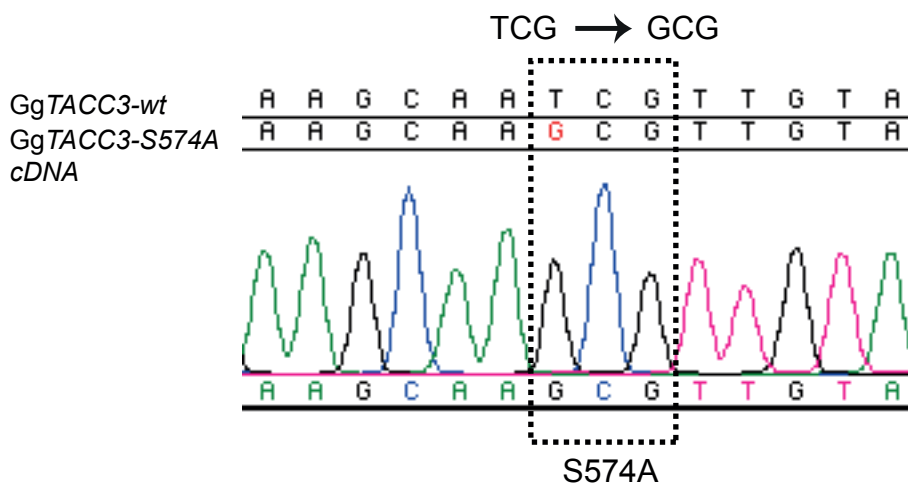

Supplement: S5 Fig — (A) S574A mutation was incorporated into exon 7 of the left arm of the targeting construct with the antibiotic resistance cassettes flanked by LoxP sites (triangles) introduced into intron 8. (B) Confirmation of gene targeting events by PCR using genomic DNA extracted from WT, S574A- heterozygous and S574A- homozygous cell lines. Block arrows show the position of primers. The antibiotic resistance cassettes were removed by Cre recombinase mediated excision. (C) Sequencing of cDNA prepared from the homozygous TACC3-S574A DT40 cells confirmed the incorporation of the mutation in to the genomic locus. (PDF) [file pgen.1005345.s005.pdf]

**Figure S6.**

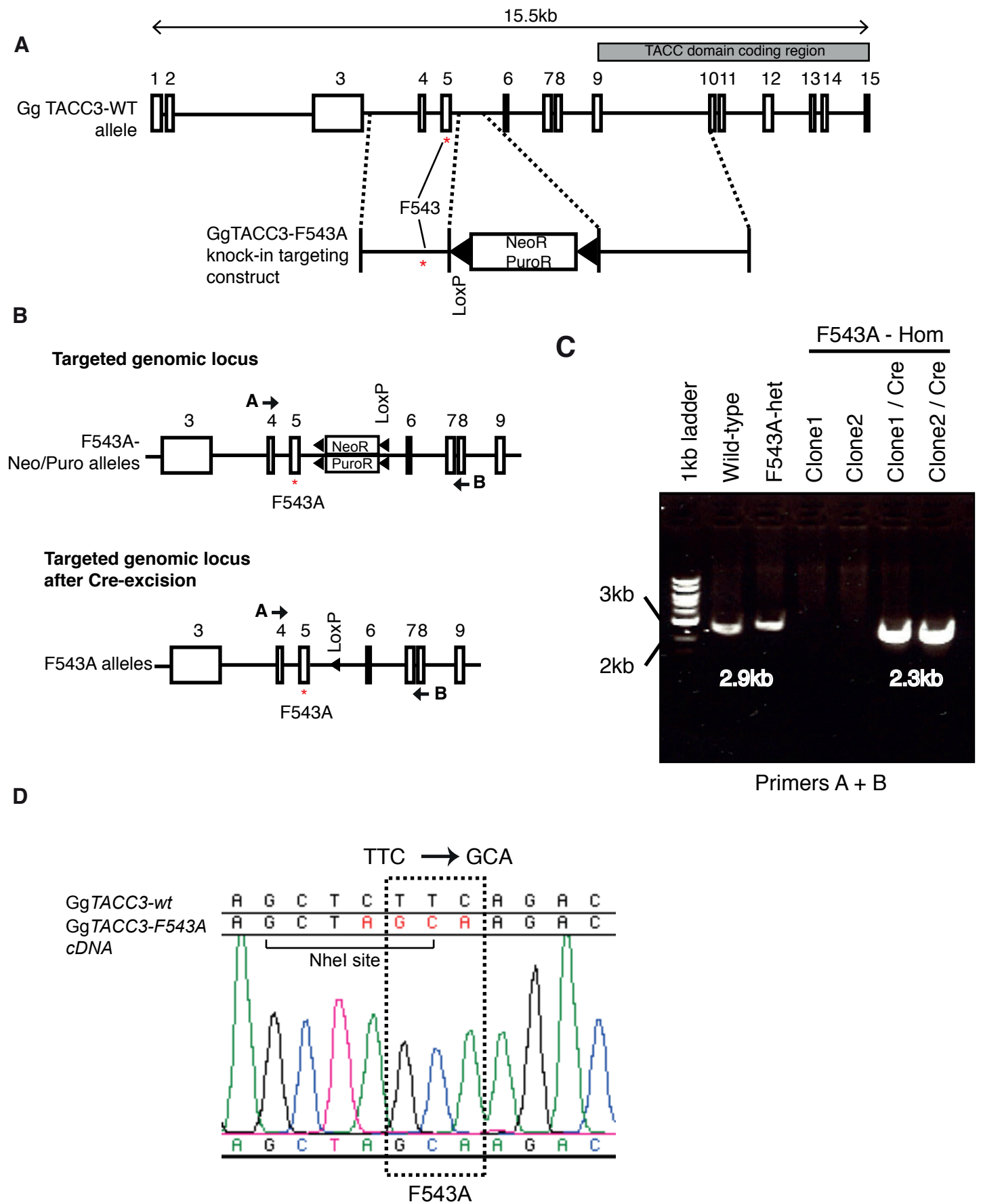

Supplement: S6 Fig — (A) and (B) F543A mutation was incorporated into exon 5 of the left arm of the targeting construct with the antibiotic resistance cassettes flanked by LoxP sites (triangles) introduced into intron 5. (C) Confirmation of gene targeting events by PCR using genomic DNA extracted from WT, F543A- heterozygous and F543A- homozygous cell lines. Block arrows show the position of primers. The antibiotic resistance cassettes were removed by Cre recombinase mediated excision. (D) Sequencing of cDNA prepared from the homozygous TACC3-F543A DT40 cells confirmed the incorporation of the mutation into the genomic locus. (PDF) [file pgen.1005345.s006.pdf]

**Figure S7.**

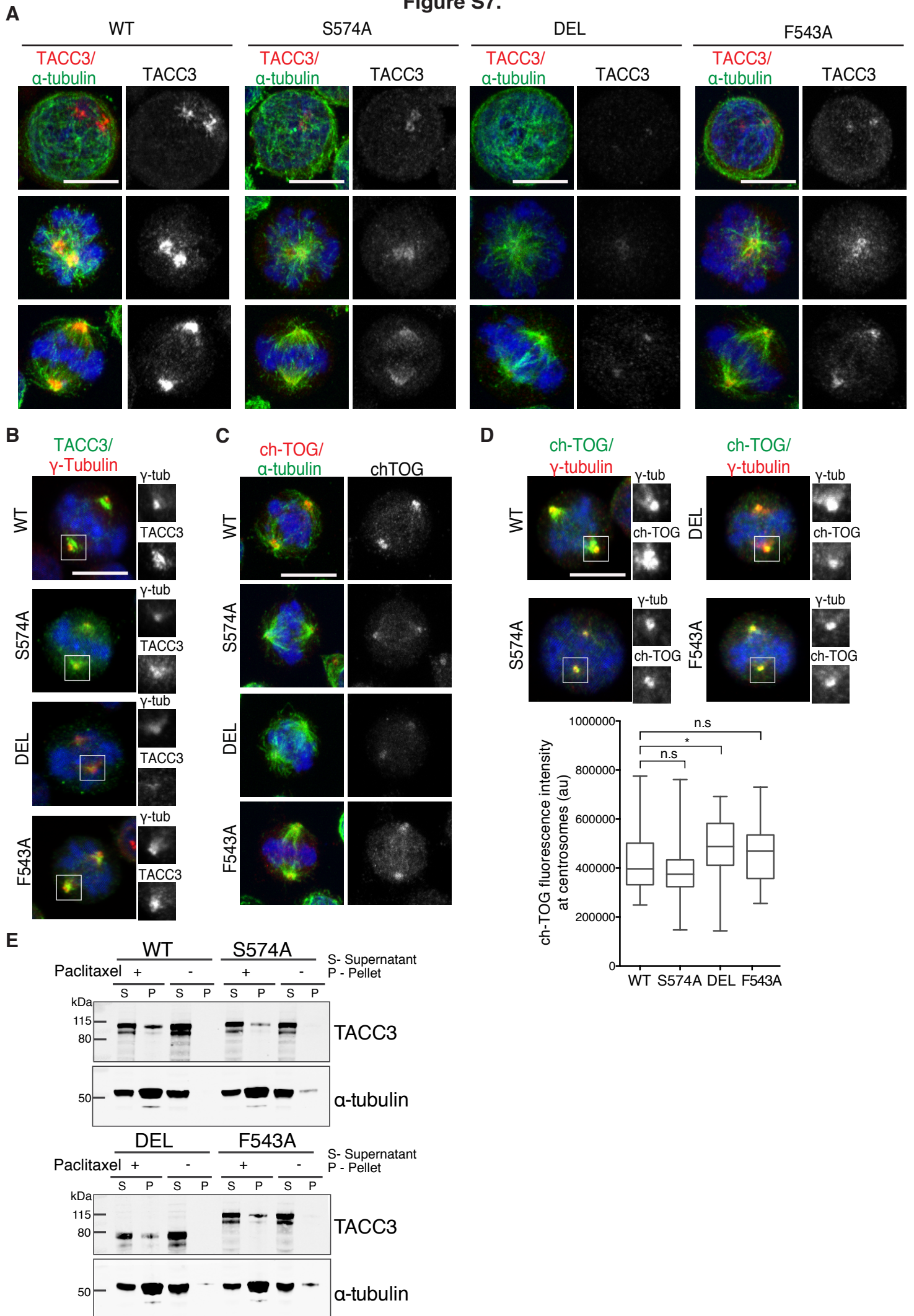

Supplement: S7 Fig — (A) Anti-TACC3 antibody staining is shown in DT40 cells of various genotypes in G2 (top panels), prometaphase (middle panels) and metaphase (bottom panels). In merged images TACC3 is in red, α-tubulin is green and DNA is blue. (B) TACC3 localisation with respect to the centrosome is shown in DT40 cells of various genotypes. Centrosomes are marked by anti-γ-tubulin antibodies in red, TACC3 is green and DNA is blue. Framed areas are shown at higher magnification below. Note that TACC3-DEL localises weakly to MT minus ends, but not to centrosomes. (C) Levels of the MT polymerase, ch-TOG, are reduced on the mitotic spindle in the TACC3 mutant lines. The extent of ch-TOG reduction correlates with the degree of TACC3 loss from the spindle (Fig 5E) with F543A being the mildest. In merged images α-tubulin is green, ch-TOG is red and DNA is blue. (D) ch-TOG remains associated with the centrosome in the TACC3 mutant cell lines. In merged images γ-tubulin is red, ch-TOG is green and DNA is blue. Box plot depicts overall intensity of ch-TOG staining at centrosomes. Ch-TOG signal intensity was quantified in centrosome volumes defined by γ-tubulin staining. A minimum of 50 centrosomes was scored per genotype. Whiskers in box plot correspond to minimum and maximum values and the boxes to the interquartile range for each genotype. Statistical significance was assessed using Mann Whitney nonparametric t-test (* P < 0.05). Scale bar = 5 μm. (E) MT-pelleting experiments were performed using mutant cell extracts as indicated. MT polymers were obtained by incubating purified tubulin with paclitaxel. (PDF) [file pgen.1005345.s007.pdf]

Figure S8

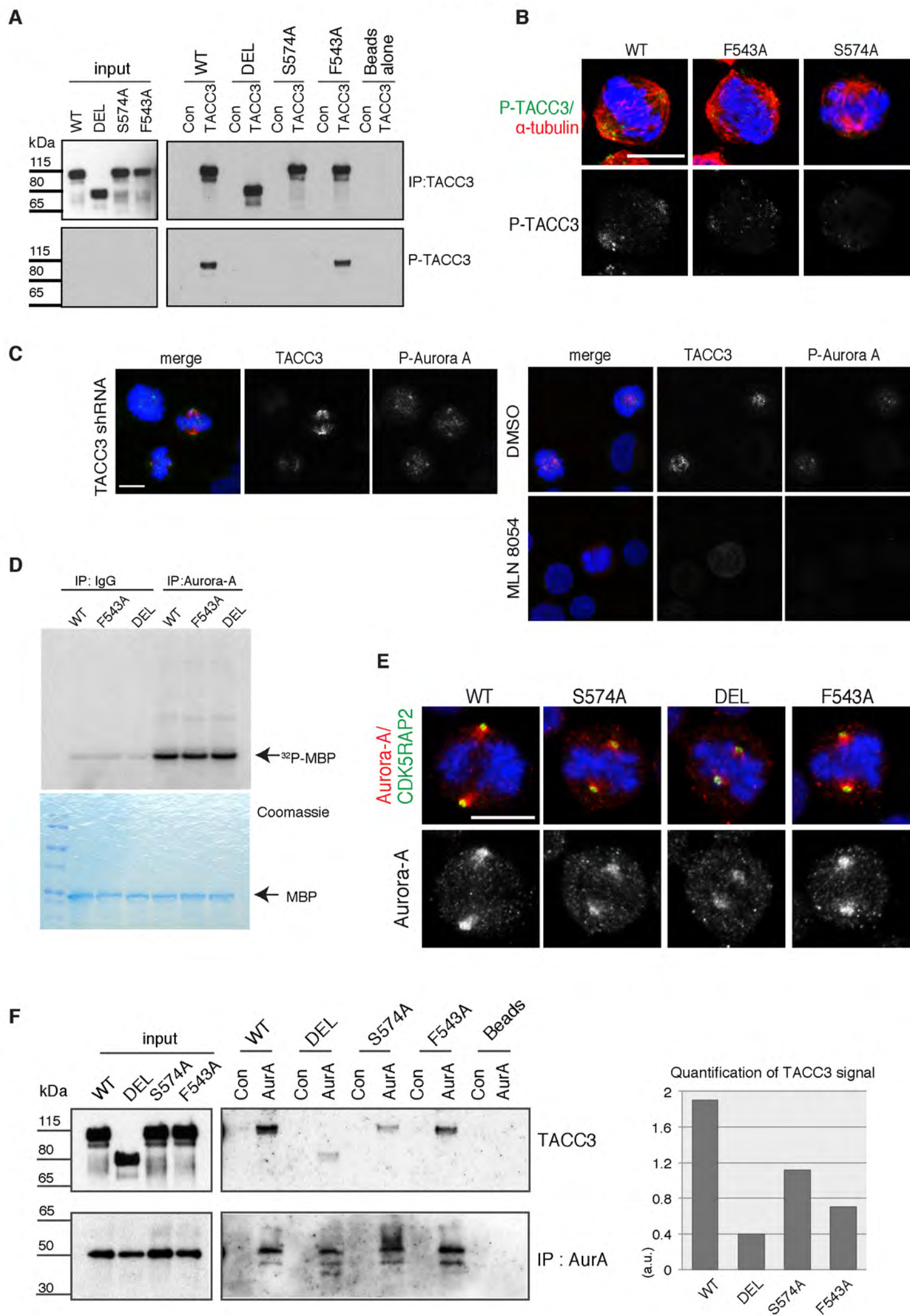

Supplement: S8 Fig — (A) Western blots show immunoprecipitation of TACC3 from DT40 cell extracts. Genotypes are as indicated. Antibodies against TACC3 or random rabbit IgG (Con) were used for immunoprecipitation (IP). Blots were probed with anti-TACC3 or anti-phospho-S574-TACC3 (P-TACC3) antibodies. Note the absence of P-TACC3 signal in S574A and DEL cells confirming the specificity of the P-TACC3 antibody. We were unable to detect P-TACC3 in the inputs. (B) TACC3F543A is phosphorylated by Aurora-A. Antibodies against phospho-S574 TACC3 (P-TACC3) weakly stain spindle poles of WT cells. Some spindle pole staining is also visible in F543A cells. Note the absence of staining in S574A cells confirming the specificity of the P-TACC3 antibody. In merged images α-tubulin is red, phospho-TACC3 is green and DNA is blue. (C) TACC3 is dispensable for T loop phosphorylation of Aurora-A. A phospho-specific antibody detects phosphorylation at T288 of Aurora A. Mitotic cells expressing varying levels of TACC3 contain similar amounts of phosphorylated Aurora-A. Note that the centrosomal signal corresponds to Aurora-A, whereas signal overlapping the chromatin is likely to reflect phosphorylated Aurora-B, as the antibody also reacts with T-loop phosphorylated Aurora-B. Lower panels show Jurkat cells after treatment with DMSO or the Aurora-A kinase inhibitor MLN8054 (4 M). The phospho-antibody is specific, since staining disappears upon treatment of Jurkat cells with MLN8054. In merged images TACC3 is red, P-Aurora-A is green and DNA is blue. (D) Aurora-A kinase immunoprecipitated from WT and TACC3 mutant cell lines shows similar activity in vitro. Aurora-A was immunoprecipitated using anti-Aurora A antibody (Aurora A IP) and random IgG was used as control (control IP). The autoradiography (32P-MBP, top panel) corresponds to the Coomassie-stained gel (bottom panel). (E) Aurora-A localizes normally in all three TACC3 mutant cell lines. In merged images the centrosomal marker CDK5RAP2 is green, Aurora-A is [file pgen.1005345.s008.pdf]

Figure S9

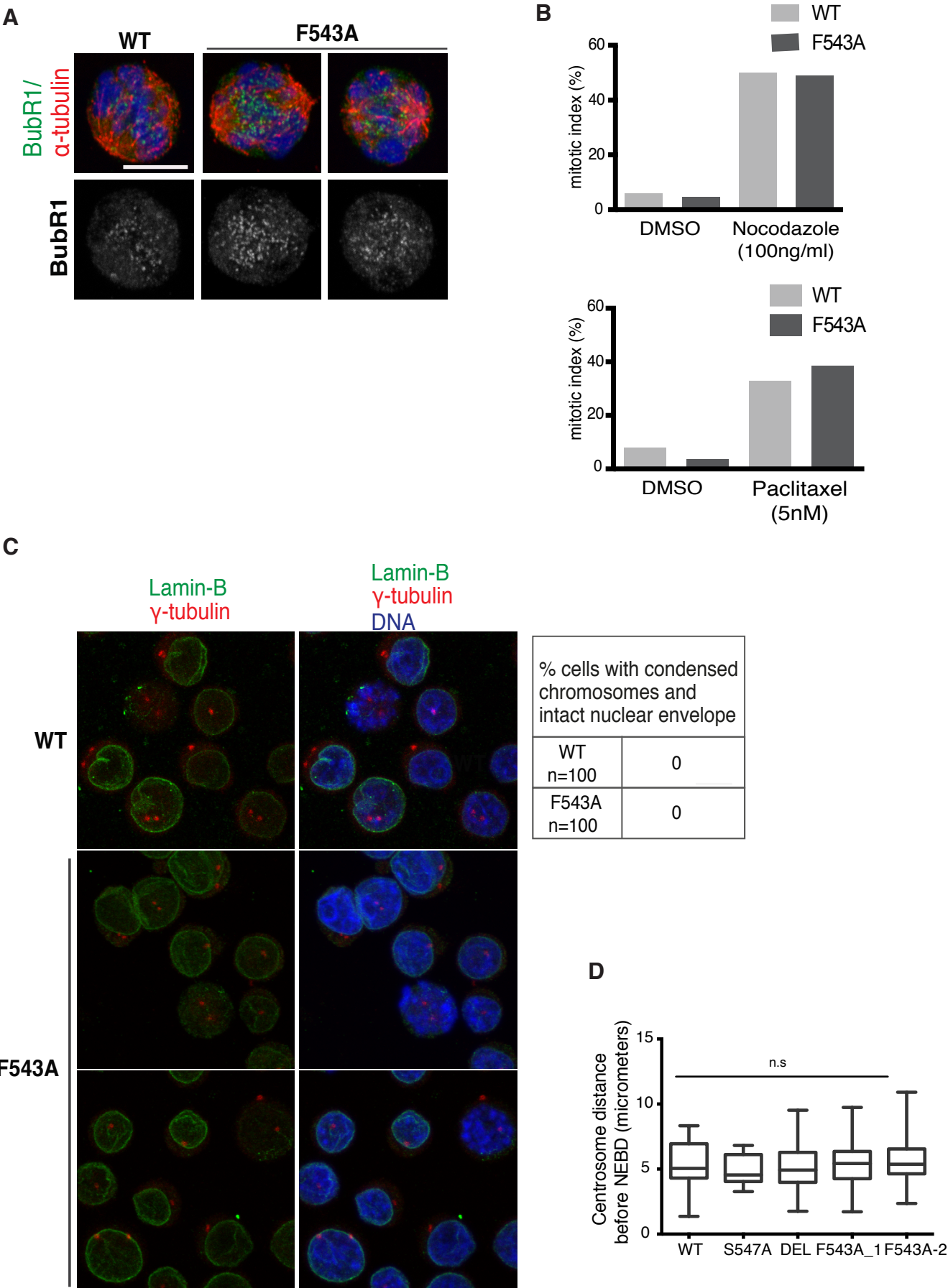

Supplement: S9 Fig — (A) Localisation of the SAC component, BubR1, to kinetochores is comparable between F543A and WT cells. In merged images k-fibres are stained with anti-α-tubulin (red), anti-BubR1 (green) and DNA (blue). (B) F543A cells arrest in mitosis as efficiently as WT. Cells treated for 6 hours with low doses of nocodazole (100 ng/ml) or paclitaxel (5 nM) were analysed. Mitotic index was quantified by Hoechst staining with >500 cells analysed. (C) Lamin B antibody reveals intact nuclear envelope in F543A cells. Lamin B is green, the centrosome marker γ-tubulin is red and DNA is blue. (D) Inter-centrosome distances in the last frame before NEBD are plotted against duration of NEBD-anaphase onset based on time-lapse experiments shown in Fig 7B and 7C. Scale bars = 5 μm. (PDF) [file pgen.1005345.s009.pdf]
