# Supplementary material for: Aurora-A-Dependent Control of TACC3 Influences the Rate of Mitotic Spindle Assembly
Source: PLoS Genet. 2015 Jul 2;11(7):e1005345. doi: 10.1371/journal.pgen.1005345 (PMC4489650; doi:10.1371/journal.pgen.1005345)
Supplement: S1 Table — (PDF) [file pgen.1005345.s010.pdf]

Table S1

**Primer**	**Alternative name**	**Sequence (5' – 3')**
Exon4_Fwd	A	TCAAGGACAGACTGATCTTCC
Exon8_Rev	B	GACTTGATGTAGGCTTCTCAG
Blasti_Fwd	C	CTCCACACAGGCATAGAGTGTCTGC
Puro_Fwd	D	CGACCGAAAGGAGCGCACGACC
Exon14_Rev	E	GGCTGCAGTTTCTGATTTAGCTTTGTTTCGTACCTGGGCG
Exon10_Rev	F	TCCATTCAAGAGCCTCCAACTC
Exon9_Rev	G	ACTAGTTCAACAGCTGCATCC
SA-LA-fwd		accgcggtggcggccGAACCTGAAGAGCTCTTCAGAC
S574A-mutR		cttcaaatacaa**CGC**TTGCTTCCTCAAAGC
S574A-mutF		gctttgaggaagcaa**GCG**TTGTATTTGAAG
SA-LA-rev		taggggatccactagCAGAAGTTCGACTTTATATAG
SA-RA-fwd		ggtaggggatcccccATGTACTTAATATTCTTGTAT
SA-RA-rev		gaattcctgcagcccCACATCAGATGAATTAAAGC
FA-LA-SalI-fwd		gtcgacGGAATAGGGAATCAAGCTTAC
FA-LA-BamHI-rev		ggatccCTAGATGGTCATTGTGGTCCT
F543A-mutF		GAACCTGAAGA**gctagc**AAGACCATCAGCAGAA
F543A-mutR		TTCTGCTGATGGTCTT**gctagc**TCTTCAGGTTC
FA-RA-BamHI-fwd		ggatccCTGCACTTACACGTGTGTCTT
FA-RA-NotI-rev		gcggccgcCCATTCAAGAGCCTCCAACTC
DEL-LA-KpnI-fwd		ggtaccTCTCTTCCACTGGATAATAC
DEL-LA-BamHI-STOP-rev		ggatcc**CTA**AGTGCTAGCTGTCTGCTGATC
DEL-RA-BamHI-fwd		ggatccGACGCTATTATAGATGTGCTG
DEL-RA-NotI-rev		gcggccgcCTAGCCAGATATTCTTCAGCA

**Human TACC3 shRNA oligos:** (5' – 3'); target sequence is shown in bold	
TACC3-shRNA2	ctcgagAAGGTATATTGCTGTTGACAGTGAGCGAAGGAAGTTCTGAGAACCAAAT tagtgaagccacagatgta**ATTTGGTTCTCAGAACTTCCTG**TGCCTACTGCCTCGgaattc
TACC3-shRNA3	ctcgagAAGGTATATTGCTGTTGACAGTGAGCGCTCCAGAAAGTTCTAAAAGAAA tagtgaagccacagatgta**TTTCTTTTAGAACTTTCTGGAT**TGCCTACTGCCTCGgaattc
